# Supplementary material for: Active disambiguation guides inferring controllability and cause in social interactions
Source: Nat Commun. 2025 Dec 22;16:11568. doi: 10.1038/s41467-025-67853-8 (PMC12748874; doi:10.1038/s41467-025-67853-8)
Supplement: Supplementary file 1 — Supplementary information [file 41467_2025_67853_MOESM1_ESM.pdf]

SUPPLEMENTARY INFORMATION

Active disambiguation guides inferring controllability and cause in social interactions

Lisa Spiering*, Hailey A Trier, Jill X O'Reilly, Nils Kolling, Marco K Wittmann, Matthew F S Rushworth† and Jacqueline Scholl†

Supplementary figures

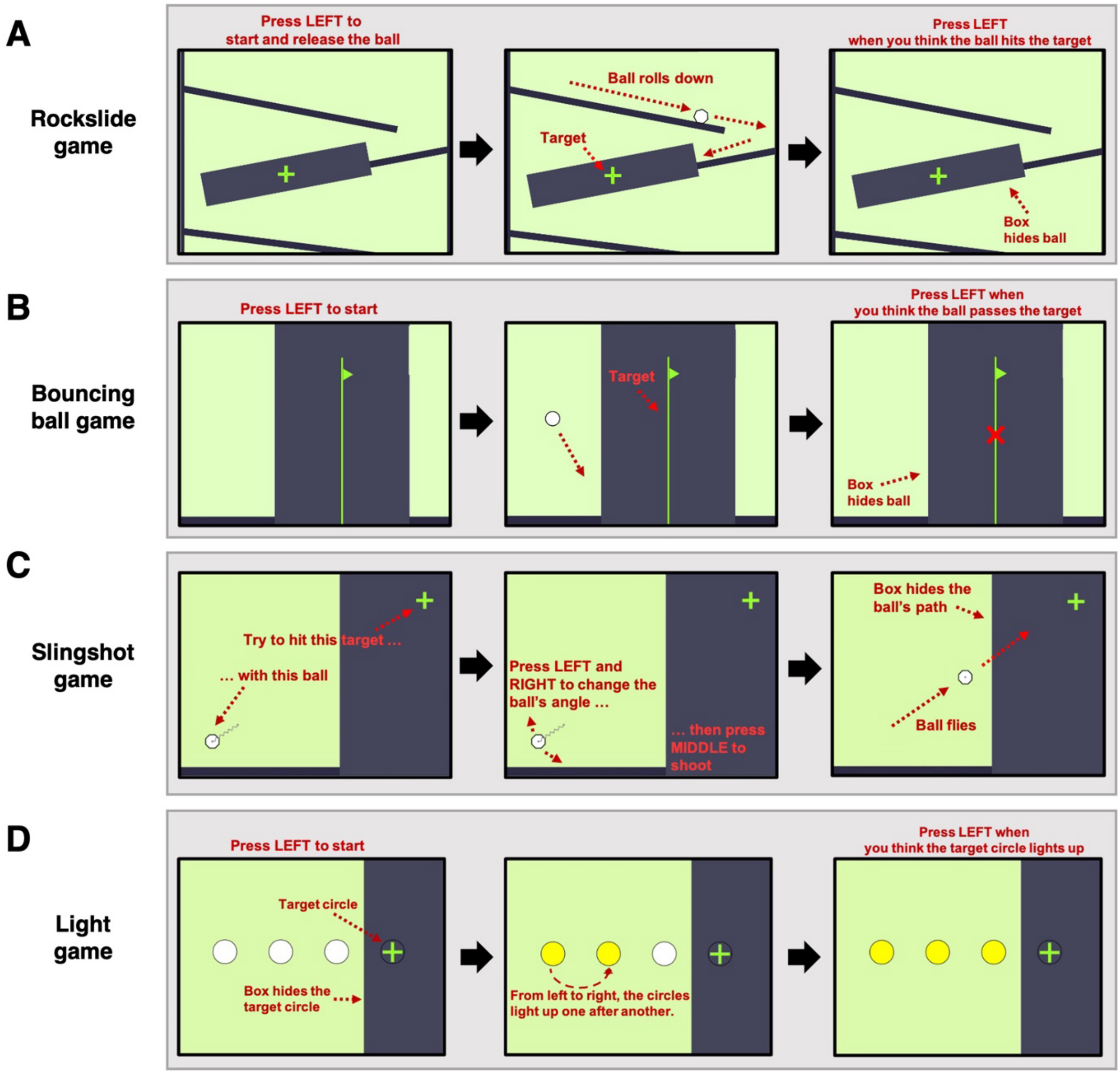

Supplementary Figure 1 (relating to Figure 1). Games in the credit assignment task. A) In the Rockslide game, participants had to press the left button to release a ball that then rolled down a set of slides from the top. Participants then pressed the left button again to indicate when they thought that the ball passed the target cross. A black box hid the ball's path so that they could not see when the ball passes by the target exactly. The box was centred around the target cross. From trial to trial, the target cross changed its position slightly on the second slide. **B)** In the Bouncing ball game, participants pressed the left button to release a ball that would appear from the left and bounce towards the right. Participants pressed the left button again when they thought the ball passed the target flag. The black box hid the ball's path so that participants again were not able to tell when exactly the ball passed the target flag. On every trial, the target flag had a different horizontal position and the black box was centred around the target flag. **C)** In the Slingshot game, participants adjusted the angle of a ball (with left and right buttons) to hit a target cross. They pressed their middle button to shoot the ball. Again, a black box hid the ball's path so that participants were not able to see how accurately they shot the ball. On every trial, the target cross changed position vertically. **D)** In the Light game, the participants saw a set of white circles. When participants pressed the left button to start, the white circles lit up one after another from the left to the right. The fourth circle, the target circle, was hidden behind a black box so that participants did not see when it lit up. They had to press the button when they thought that the target circle would light up. On every trial, the latency between start (when participants pressed the left button the first time) and when the target circle would light up changed.

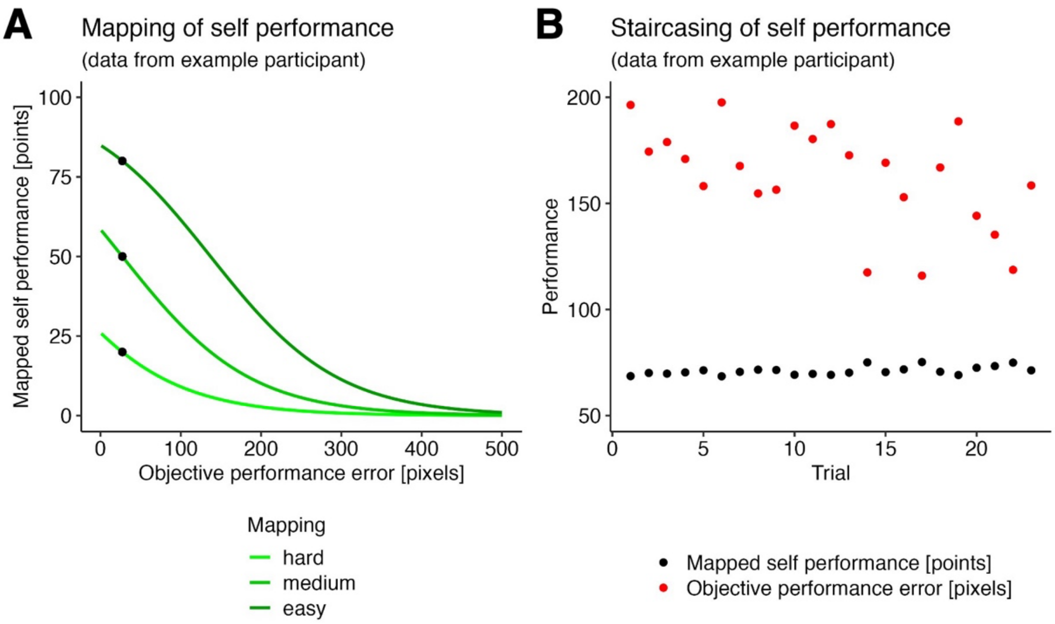

Supplementary Figure 2 (relating to Figure 1). Transformation of objective game performance into self performance. A) Mapping of objective performance error onto the self performance. We used a logistic function to transform participants' objective performance errors into the self performance. A lower objective performance error (i.e. higher accuracy) resulted in higher self performance. Note that under different mapping conditions, the same objective performance error resulted in differently mapped self performances (black dots). Values of an example participants are shown here. **B)** Staircasing of self performance. Over time, this example participant drifted in their objective performance (red dots) and got better over time. By using a staircasing procedure, we transformed their drifting performance into a stable self performance (black dots). This meant that even if participants objectively got better or worse at the games over time, the mapped self performance remained the same.

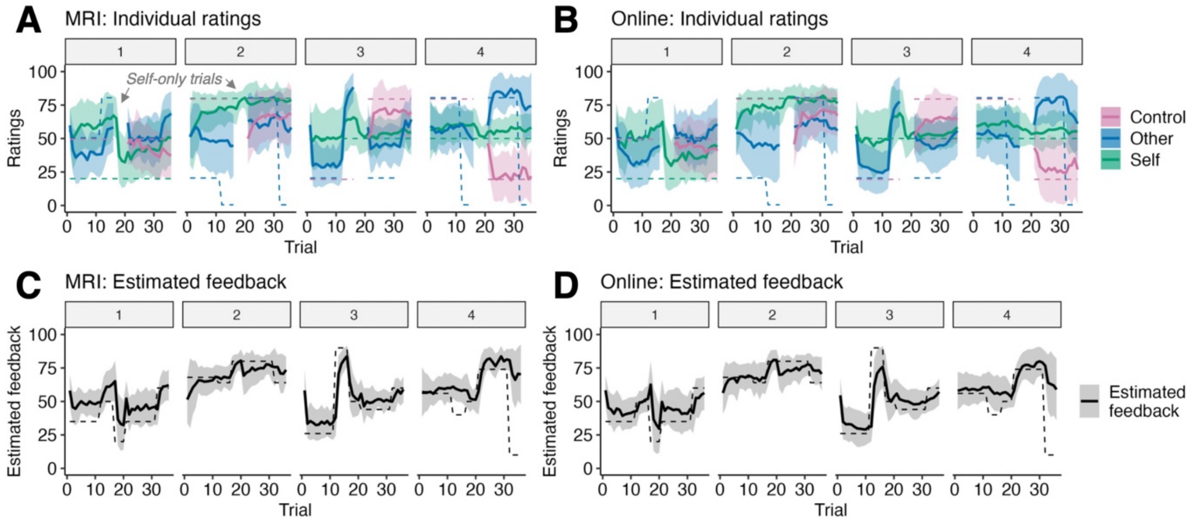

Supplementary Figure 3 (relating to Figure 2B-C). Participants' average ratings and estimated feedback in each of the four task blocks. Shaded intervals are SD. Individual ratings of the **A)** MRI sample and **B)** Online sample. During the Self-only trials, when the feedback reflects only the self and participants only rated their own performance, their self estimates show a sudden adaptation to the true self level. Estimated feedback of the **C)** MRI sample and **D)** Online sample.

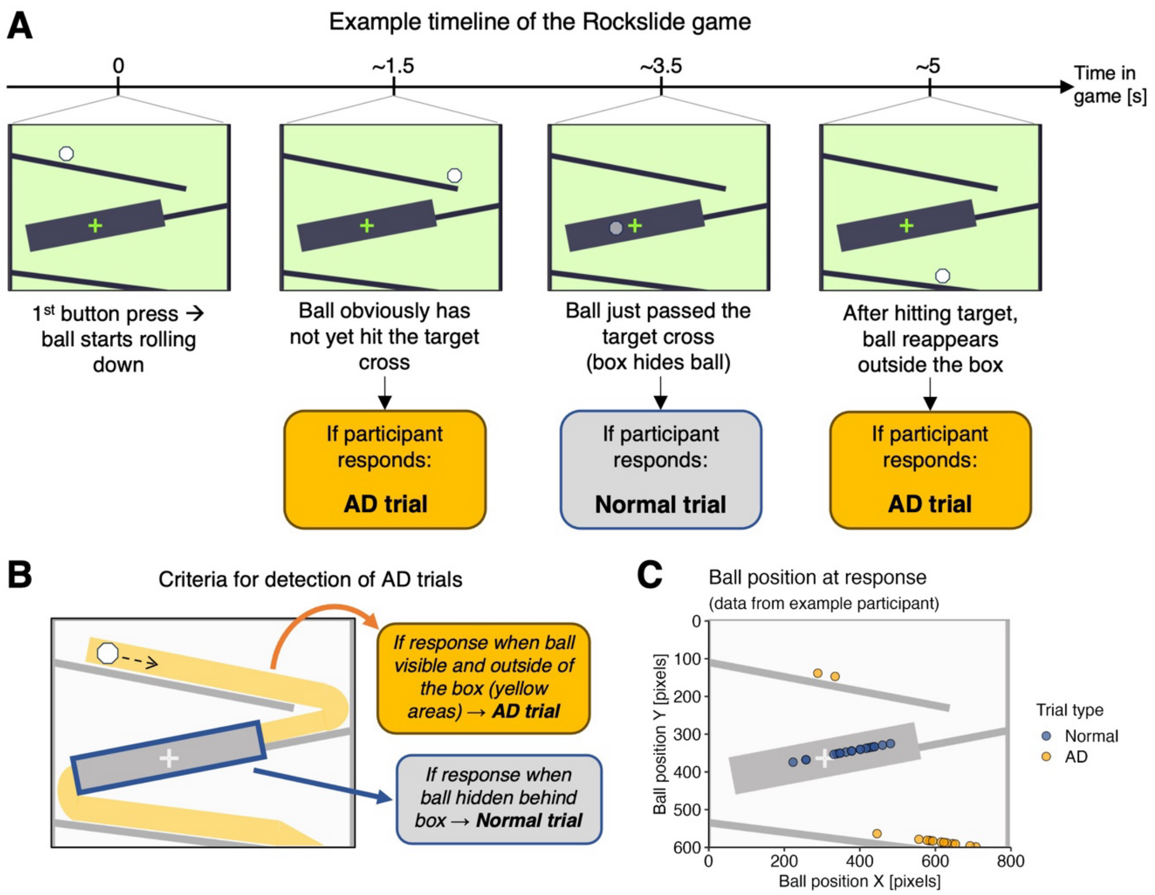

Supplementary Figure 4 (relating to Figure 3). **Detection of AD trials when participants play the games. A)** Example timeline in the Rockslide game. The game starts when participants press the button, which releases a ball that rolls down a set of slides. The black box hides the ball's path. Here, for illustration, the ball's outline is shown in the third panel but to the participant, the ball was not visible while behind the box. Depending on when participants respond (by button press), the trial is detected as normal or AD. Trials are detected as AD trials if participants make 'obvious' mistakes in the games. **B)** Specifically, this is the case if participants respond when the ball is still visible and outside of the box that hides the target cross. The trial is detected as a normal trial if participants respond when the ball is close to the target cross and hidden behind the box. **C)** Data from an example participant shows the clear difference between ball positions in AD and normal trials. Data is overlaid with an example screenshot of the rockslide game. Note that the blue dots (ball position on normal trials) vary in their positions because between trials, the target cross changed its position on the 2nd slide and participants could not see the ball behind the black box (and therefore were sometimes too early or too late in pressing the button).

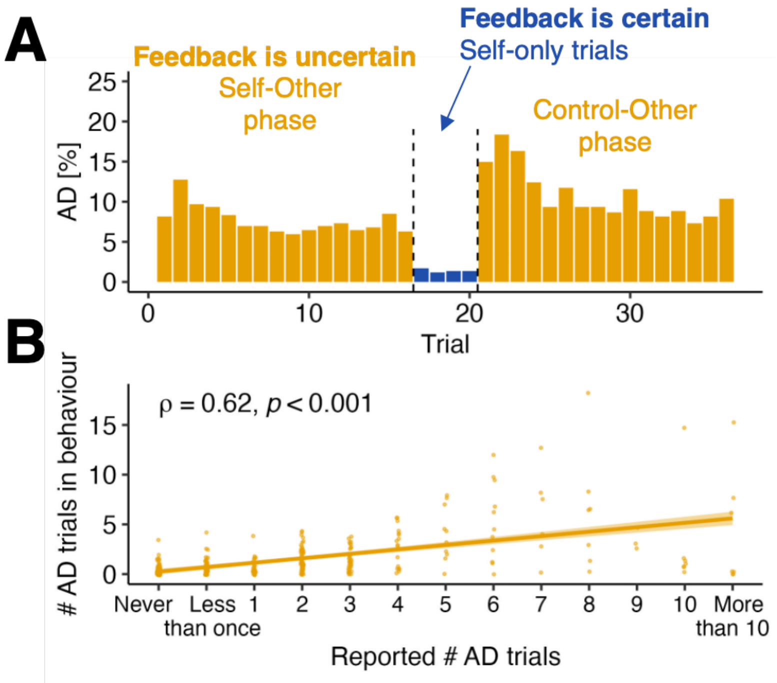

Supplementary Figure 5. AD behaviour in a large online sample (n=352 included after data quality checks) without the task hint to change game behaviour. We collected a version of our task online in which participants did not receive the hint that they can change their game behaviour during the instructions. This was part of another study and the findings will be reported elsewhere. This sample is included here to address a reviewer question about the degree to which AD behaviour is a generalizable phenomenon that does not depend on task instructions. **A)** Firstly, we found that these participants who were not primed to do AD still performed AD but they showed a lower proportion of AD compared to the primed participants reported in our manuscript (proportion of AD, averaged across Self-Other and Control-Other phases: unprimed online sample, 9.28%; [primed] online sample, 19.62%; [primed] MRI sample, 27.39%). Consistent with the findings reported in our manuscript, we found that the proportion of AD shows a strong drop when the feedback was not ambiguous and only reflected participants' own performance. Similar to our previous analysis (Figure 3c), we only included online participants for who we detected AD trials in their behaviour and who reported doing AD in the debrief questionnaire. This left us with n=147 online participants for this analysis. **B)** Secondly, we again found that the unprimed participants had insight into AD because the reported number of AD trials correlated with the number of AD trials we detected in their behaviour (Spearman's ρ=0.62, p<0.001). Here, we analysed the full online sample including participants who reported never having done AD and for who we did not detect AD (i.e. n=352 participants included). Overall, these results suggest that even though not priming participants to do AD indeed reduces AD behaviour in our task, participants still did AD and we found that this is modulated by uncertainty and that they had insight into using this strategy.

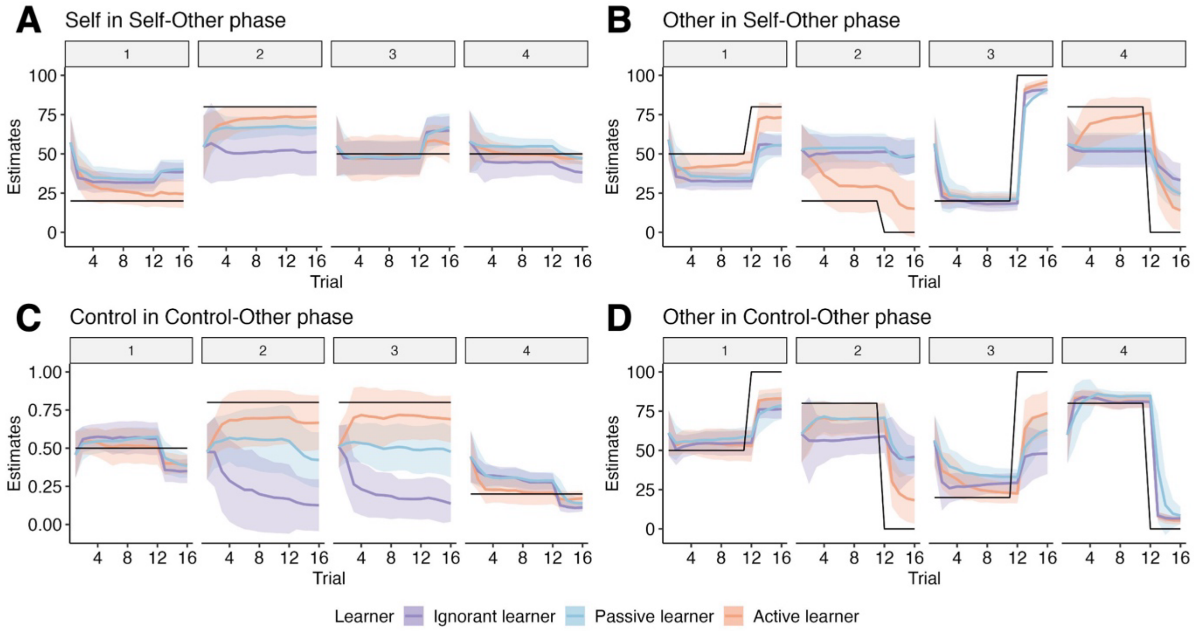

Supplementary Figure 6 (relating to Figure 3). Average estimates per game block for the active, ignorant and passive learning models. Simulated data from MRI and online sample are pooled here. Lines are averaged estimates across participants, and shaded intervals are the standard deviation. Black lines indicate the true levels of each variable. **A)** Self estimates per game block in the Self-Other phase. **B)** Other estimates in the Self-Other phase. **C)** Control estimates in the Control-Other phase. **D)** Other estimates in the Control-Other phase. The active learner has lower rating errors than the passive and ignorant learning models which make wrong assumptions about AD (Main effect of learning model, $F(2,130)=447.24$, $p<0.001$, $\eta^2=0.83$; active vs. passive learner, $F(1,65)=461.54$, $p<0.001$, $\eta^2=0.72$; active vs. ignorant learner, $F(1,65)=485.83$, $p<0.001$, $\eta^2=0.86$; passive vs. ignorant learner, $F(1,65)=275.22$, $p<0.001$, $\eta^2=0.60$). Additionally, the active learner also has the lowest uncertainty at the end of the phases compared to the other learning models (Main effect of learning model, $F(2,130)=325.07$, $p<0.001$, $\eta^2=0.79$; active vs. passive learner, $F(1,65)=350.98$, $p<0.001$, $\eta^2=0.79$; active vs. ignorant learner, $F(1,65)=351.19$, $p<0.001$, $\eta^2=0.72$; passive vs. ignorant learner, $F(1,65)=94.04$, $p<0.001$, $\eta^2=0.38$). Crucially, these results suggest that a learner, given the knowledge of AD trials and the ability to learn from it, performs better (in terms of errors and uncertainty) than the two learners which either discard AD trials for learning (passive learner) or assume that AD trials are normal trials (ignorant learner). Summed rating errors and average uncertainty were extracted from the last phase trial of each phase in each block, and averaged across the individual ratings.

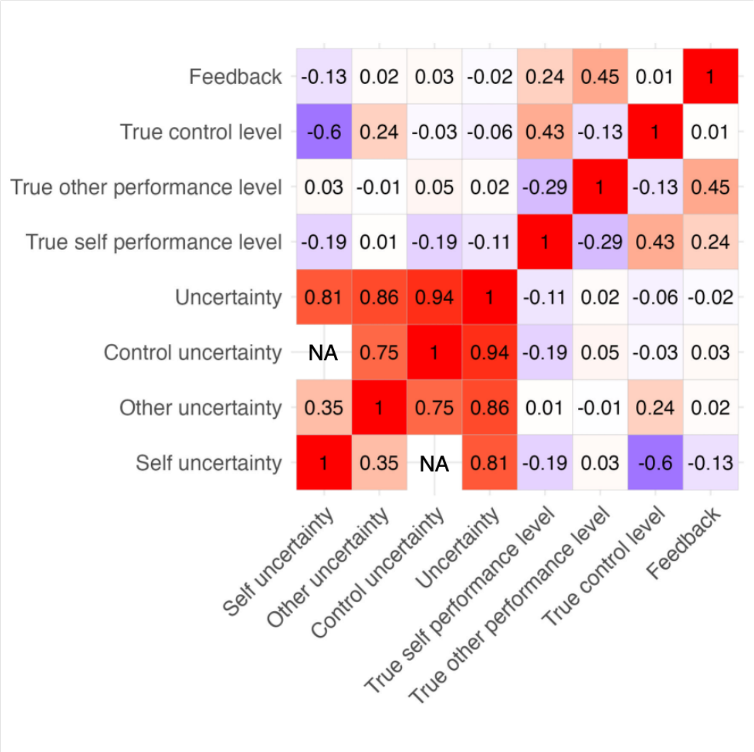

Supplementary Figure 7. Correlation matrix between the task schedule of true self, other and control levels, the feedback observed by participants, and the uncertainties extracted from the Active learning model. The true levels of control, self and other are unrelated to their respective uncertainties (absolute rs < 0.2). Data from both MRI and online samples is included.

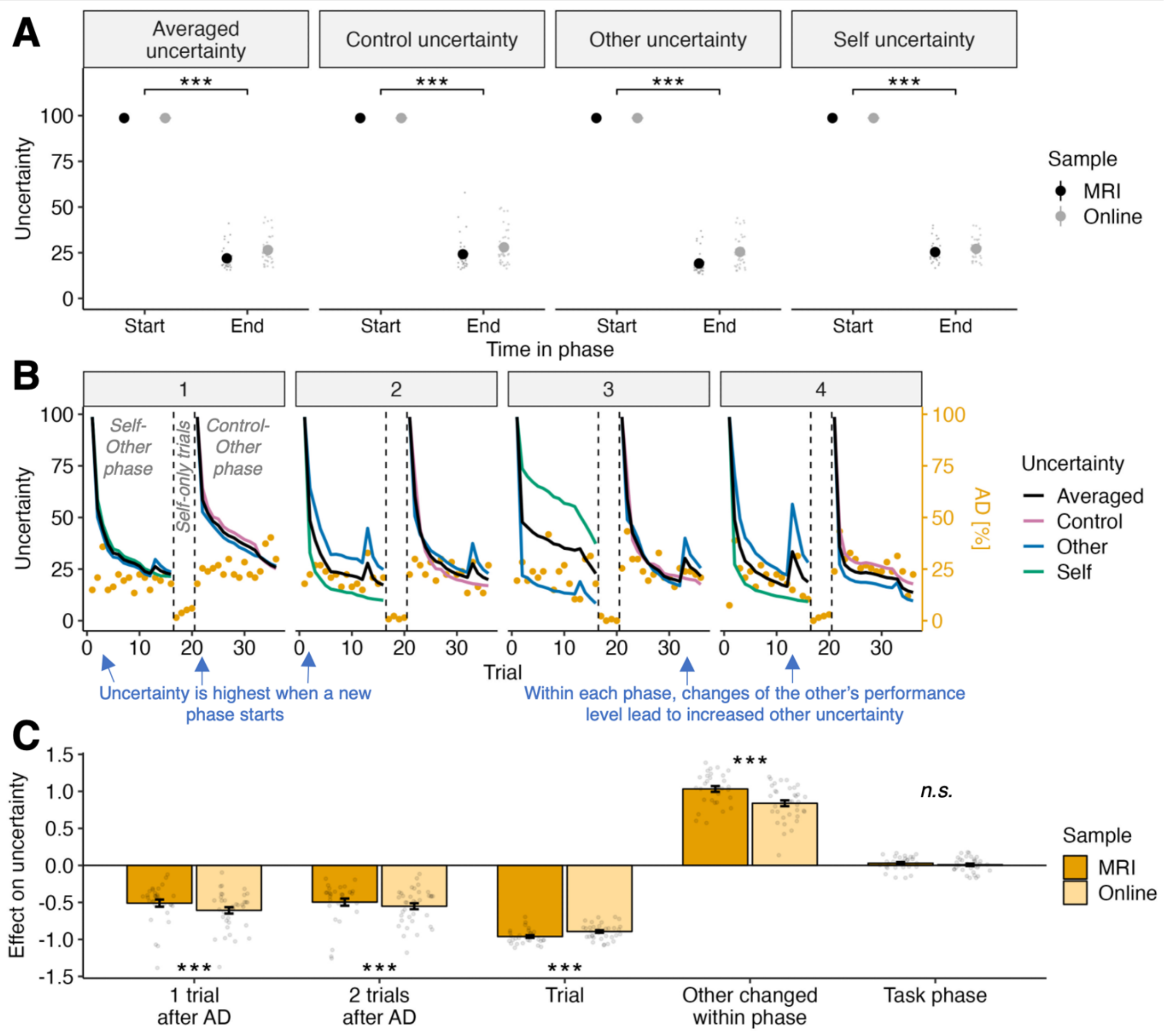

Supplementary Figure 8. The dynamics of uncertainty (extracted from the Active learner model). A) From the start to the end of a phase, uncertainty reduced (averaged uncertainty, $F(1,65)=7103.74$, $p<0.001$; control uncertainty, $F(1,65)=3807.87$, $p<0.001$; other uncertainty, $F(1,65)=6249.37$, $p<0.001$; self uncertainty, $F(1,65)=10184.59$, $p<0.001$). To test whether uncertainties reduced over time, here we plot the equivalent of Figure 2d but for uncertainties rather than rating accuracies. For each type of uncertainty (averaged, control, other and self) as dependent variable, we ran an ANOVA with *time in phase* ('Start' or 'End') as within, and *sample* (MRI or online) as between participant factors. At the start of each phase, the Active learner had the same wide uncertainty for each unknown variable and each participant. Therefore, each individual data point for 'Start' has the same uncertainty value in the plot and is covered by the bigger data point denoting the mean. Additionally, this plot and Figure 2d show the standard error of the mean, which in both cases is so small that the bigger data point denoting the mean hides the error bars. **B)** Uncertainties plotted for each game block and by task phase, averaged across Active learner model fit to MRI and online sample. While all uncertainties are highest at the start of each task phase, other and therefore averaged uncertainties increased when the other player changed performance unannounced. For reference, yellow dots indicate the proportion of AD trials, averaged across MRI and online samples. **C)** Full results plot of the regression shown in Figure 3g in the main text. (Averaged) uncertainty was reduced on trials following AD (as reported in main text), and uncertainty reduced over time within a phase (trial, $F(1,65)=5813.88$, $p<0.001$, $\eta^2=0.99$). Uncertainty increased when the other's performance changed unannounced during the task phase (other changed within phase, $F(1,65)=1124.28$, $p<0.001$, $\eta^2=0.95$). Averaged uncertainty did not differ between task phases (task phase, $F(1,65)=2.65$, $p=0.11$, $\eta^2=0.04$).

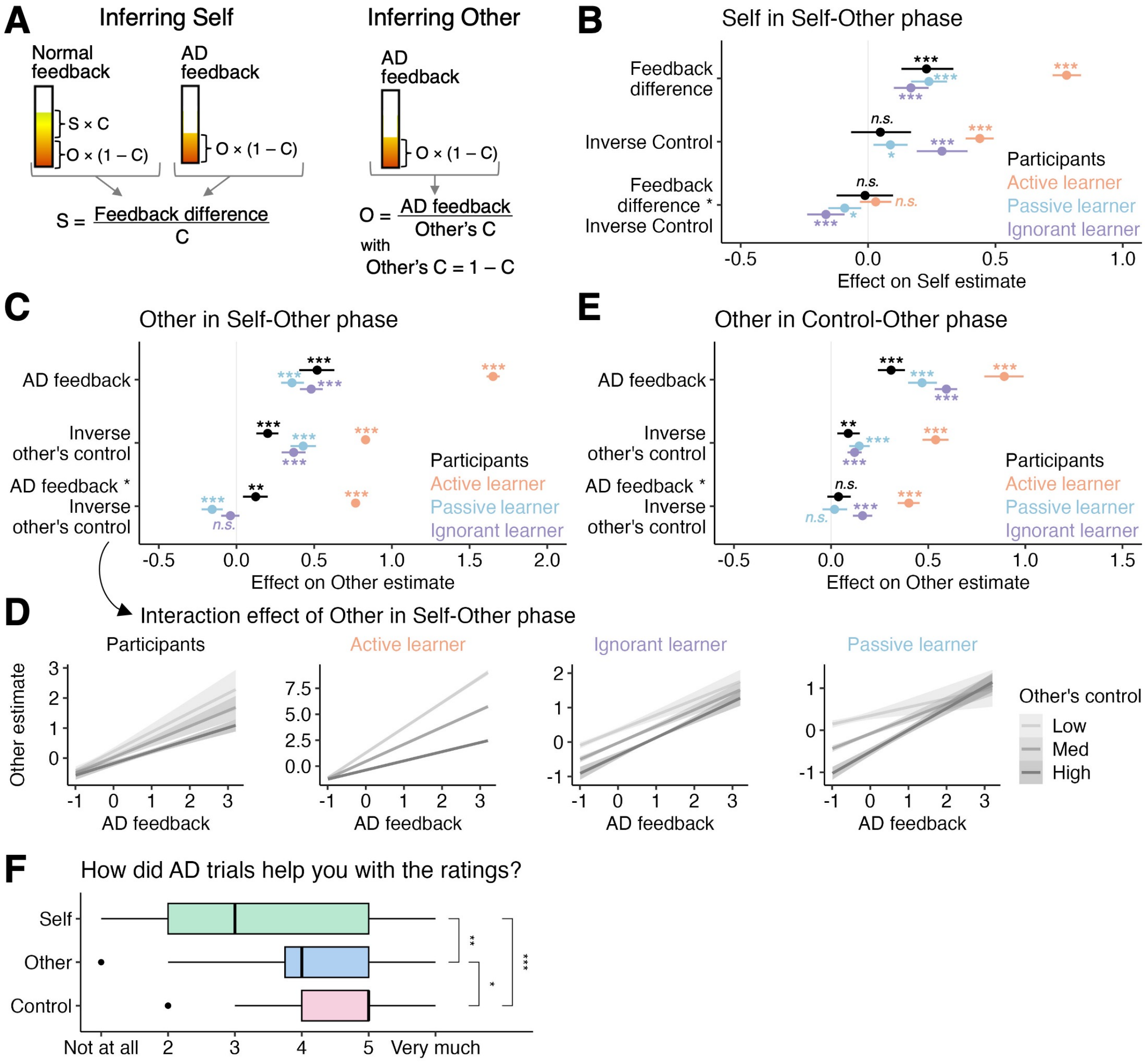

Supplementary Figure 9 (relating to Figure 4). Hierarchical regressions predicting how to assign credit from switch and AD trials to self and others. A) Optimally, in the Self-Other phase, self can be inferred from the feedback difference between normal and AD trials accounting for one's control. Note that this strategy is symmetrical to how control is inferred in the Control-Other phase (main text Figure 4). In both Self-Other and Control-Other phase, the other's performance can be inferred by just observing the AD feedback and accounting for the other's control (1-control). Note that in the Self-Other phase, the control is known while in the Control-Other phase, this requires the control which needs to be inferred as well. **B)** For inferring self performance in the Self-Other phase, we found that participant performance neither resembled the Active learner (main effect of inverse control), nor the Passive or Ignorant learning models (inverse control and interaction effect). This means that participants were not able to optimally extract the information afforded by AD and normal trials to infer their own performance. This is also reflected in their helpfulness ratings in the debrief questionnaire (panel F), where they reported AD trials as the least helpful for the self ratings. **C**) In contrast, participants inferred the other's performance similarly to the Active learner in the Self-Other phase. Here, the interaction effect AD feedback * Inverse other's control differentiated between, on one hand, participants and the active learner, and on the other hand, the two alternative learning models (which make wrong assumptions about AD). Note however that participants' beta weights are not as close to the active learner as they are for the respective control inference (Figure 4D). Participants also rated AD trials as more helpful for learning about their control than learning about the other (panel F). For these reasons, we focussed our analyses on the control inference process and did not follow up this effect in inferring the other. **D)** The interaction effect from C shows that both participants and the Active learner inferred that the other had a low performance if the AD feedback is low, irrespective of the other's control. If the AD feedback is high, however, they inferred a higher other performance level and even more so if the other had a low control because then the other's performance must account for the high AD feedback. The passive and ignorant learners show markedly different behaviour in this interaction effect. Note that for easier interpretation, here, we relabelled the legend of "inverse other's control" regressor to "Other's control" and flipped its levels accordingly (e.g. "Low other's control" is the effect of "high inverse other's control"). **E)** In the Control-Other phase, we found that

participants were not able to infer the other's performance like the Active learner (see interaction effect). **F)** In the debrief questionnaire, participants reported that they found the AD trials most helpful for inferring their controllability (ANOVA main effect of rating type (self, other, or control): $F(2,70)=13.57$, $p<0.001$, $\eta^2=0.17$; paired two-sided t-tests: self vs. other, $t(35)=-3.29$, $p=0.002$, $d=0.55$; self vs. control, $t(35)=-4.50$, $p<0.001$, $d=0.75$; other vs. control, $t(35)=-2.22$, $p=0.03$, $d=0.37$). Only data from the online sample is shown here because the MRI sample did not receive this debrief question. n=31 MRI, n=36 online; panels B-E: n.s., 95% CI includes 0; *, 95% CI excludes 0; **, 99% CI excludes 0; ***, 99.9% CI excludes 0; panel F: *, p<0.05; **, p<0.01; ***, p<0.001.

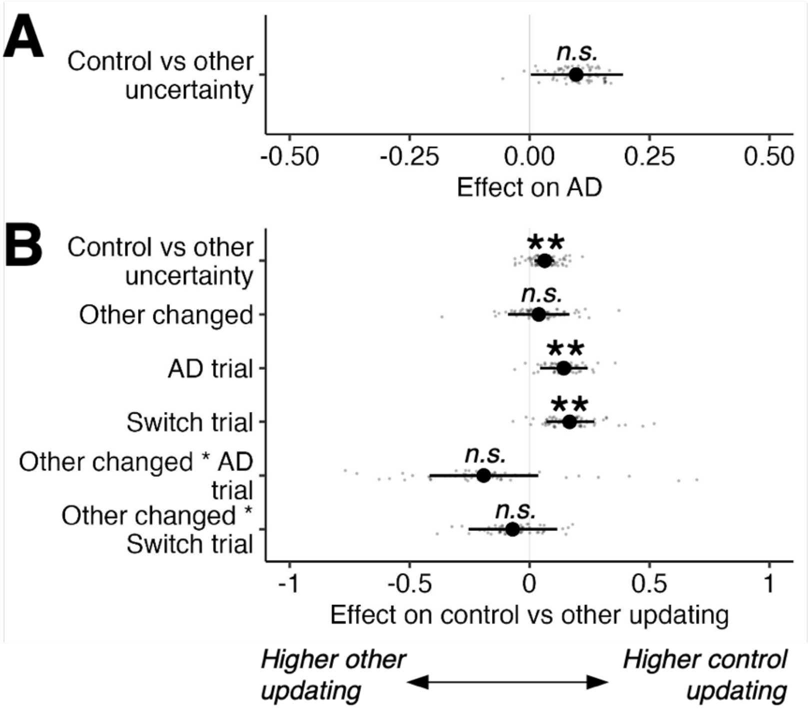

Supplementary Figure 10. Effects driving AD trials and learning in the Control-Other phase. A) Firstly, we examined whether AD trials were driven more by the uncertainty about control than about the other player in the Control-Other phase. Intuitively, if the difference between control vs other uncertainty has a positive effect on AD, then AD trials were more driven by control than other uncertainty. If control vs other uncertainty has a negative effect, then AD trials were more driven by uncertainty about the other player. We did not find a significant effect of the relative control vs other uncertainty on AD (control vs other uncertainty, β=0.10, 95% CI=(0.00,0.19)). However, it is noteworthy that control vs other uncertainty appears to have a (non-significant) positive trend effect on AD which is present in all participants but two. This means that AD tended to be more driven by control than other uncertainty. However, since this effect was not significant, we cannot draw firm conclusions from this analysis. **B)** As expected, we found that participants updated their beliefs about control more than the other player if the control uncertainty was higher than the other uncertainty (control vs other uncertainty, β=0.06, 95% CI=(0.02,0.10)). Next, we hypothesized that both AD and switch trials might be particularly informative to update beliefs about control. Indeed, we found that participants updated their beliefs about their control more strongly than about the other following both AD and switch trials (AD trials, β=0.14, 95% CI=(0.04,0.24); Switch trial, β=0.17, 95% CI=(0.07,0.27)). We did not find that participants learned more about the other when the other's performance changed unannounced (non-significant main effect Other changed, and the interaction terms Other changed * AD trial and Other changed * Switch trial). Overall, these results suggest that throughout the Control-Other phase, participants used AD and switch trials more to learn about their level of control than to learn about the other player. Furthermore, participants updated their beliefs about their control particularly when the uncertainty about control was higher.

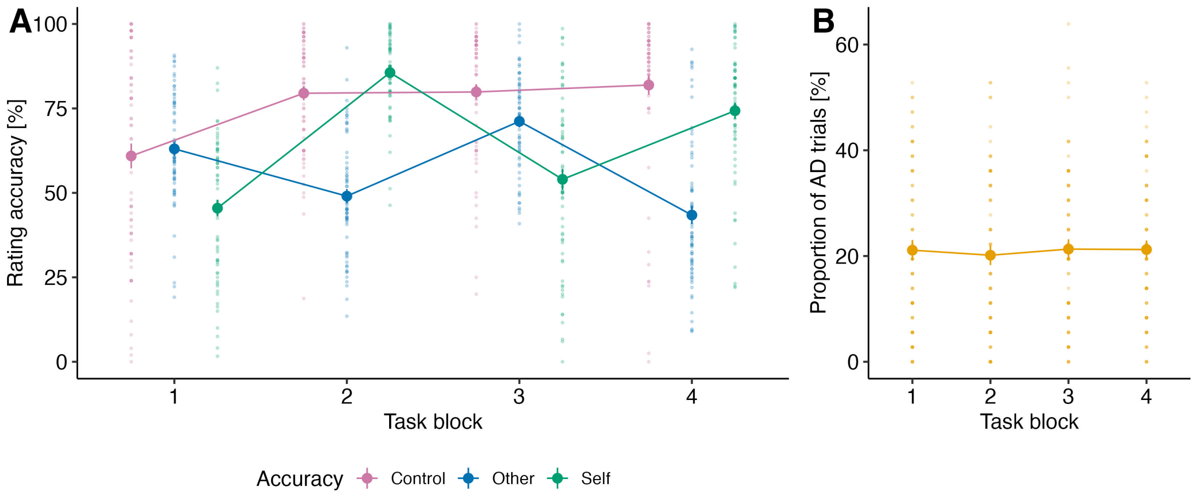

Supplementary Figure 11. Rating accuracies and AD over the course of the experiment. A) We investigated whether participants got better at learning about their own or the other player's performances over the course of the experiment. We found a significant main effect of task block on the rating accuracies of control, self and other (effect of task block on: control rating accuracy, $F(3,195)=11.36$, $p<0.001$; other rating accuracy, $F(3,195)=35.98$, $p<0.001$; self rating accuracy, $F(3,195)=59.50$, $p<0.001$). Post-hoc paired t-tests comparing the accuracies from the first to the last task block revealed that participants got better at inferring their control and their own performance, but worse at learning about the other player (control, $t(66)=4.27$, $p<0.001$; self, $t(66)=10.38$, $p<0.001$; other, $t(66)=-5.57$, $p<0.001$). In particular, the apparent steady increase of control rating accuracies from task block to task block, also compared to the noisier fluctuations of other and self accuracies, might suggest that control inference improved over the course of the task. Data from MRI and online sample are combined here. **B)** We did not find that the proportion of AD differed between task blocks (effect of task block on proportion of AD trials, $F(3,195)=0.15$, $p=0.93$), suggesting that participants did not change their AD use over time. Data from online and MRI samples are pooled here.

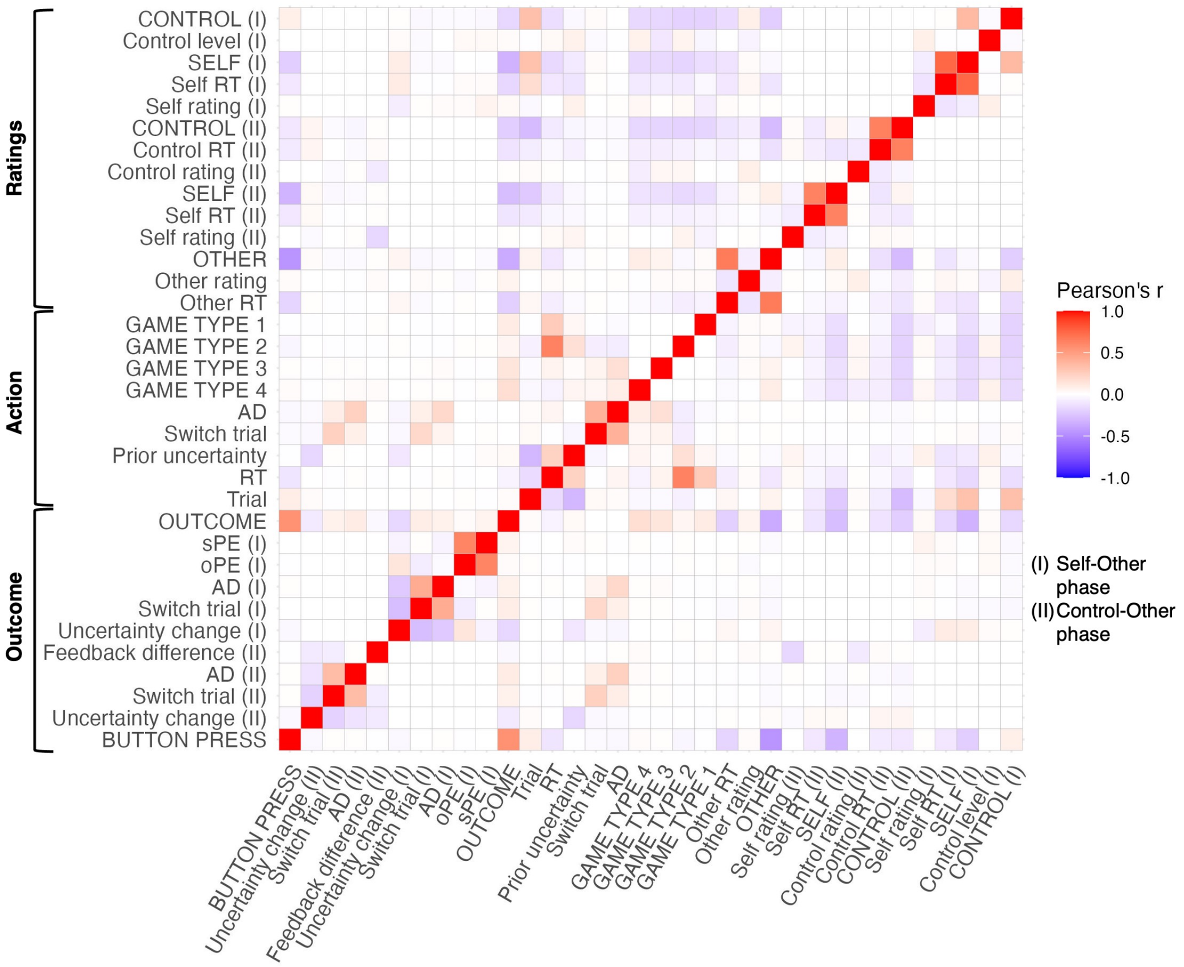

Supplementary Figure 12 (relating to Figure 5). Correlations of regressors (convolved with hemodynamic response function) included in the fMRI whole brain analysis. Pearson's correlations were averaged across participants. Constant regressors are highlighted in uppercase. In the action phase when participants play the games, we included four constant regressors, one per game type (1=Light, 2=Slingshot, 3=Rockslide, 4=Bouncing ball).

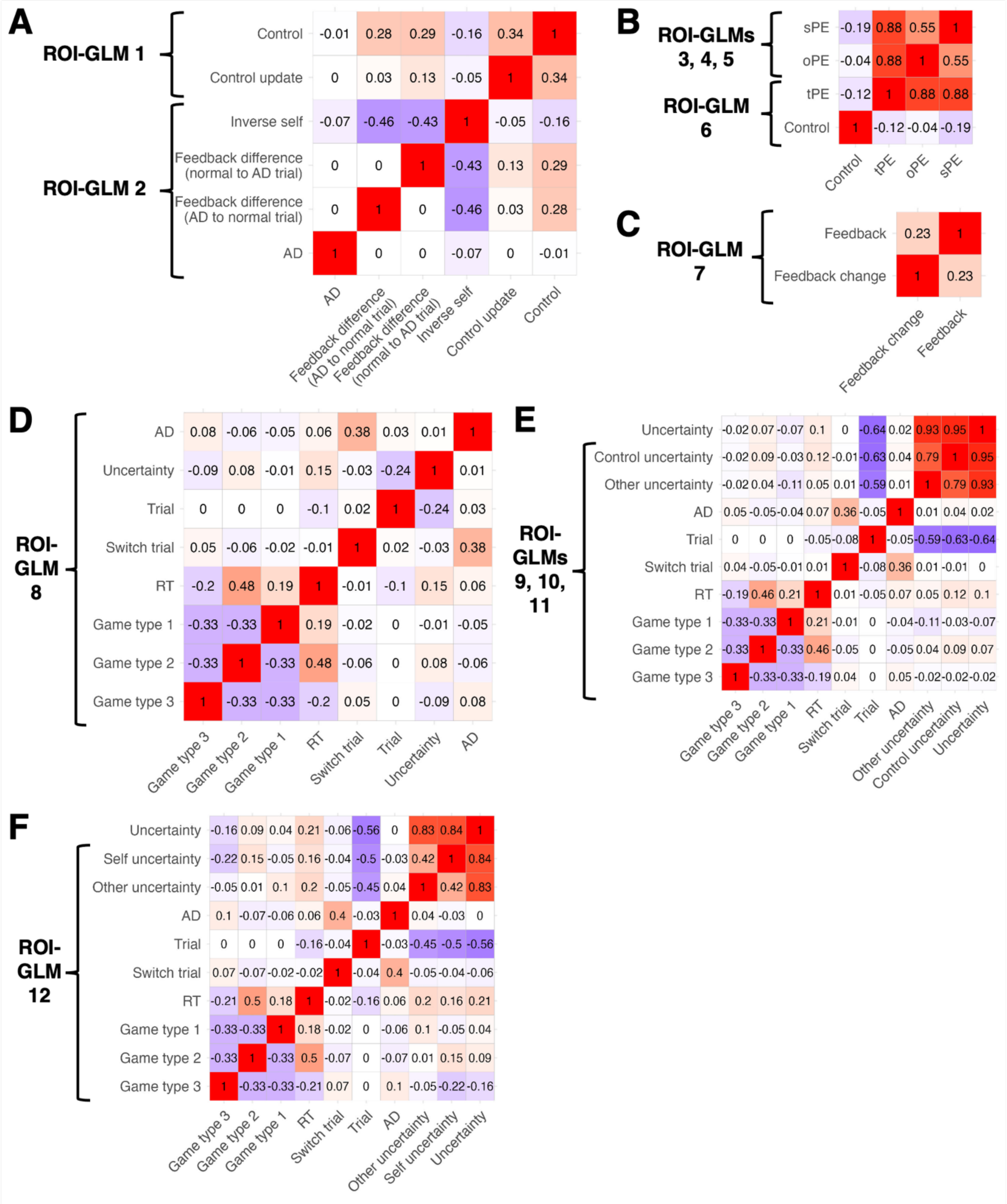

Supplementary Figure 13 (relating to Figures 6-7, Supplementary Figures 14-19). Correlation matrices for regressors included in time course analyses (ROI-GLMs). **A)** Correlation of regressors shown in main Figure 6. Regressors for the ROI-GLMs 1 and 2 are shown in one correlation matrix to highlight that the regressors of control belief (ROI-GLM 1) and feedback differences (ROI-GLM 2) show low correlations (r<0.3). **B)** Correlations of regressors shown in main Figure 7. Since sPE and oPE showed a correlation of r=0.55, we tested the time courses of these regressors first separately (ROI-GLMs 3 and 4), and then together (ROI-GLM 5). We also tested the effects of tPE and control separately in ROI-GLM 6. **C)** Regressor correlations for Supplementary Figure 17 (ROI-GLM 7). **D)** Regressor correlations for Supplementary Figures 18a and 19a (ROI-GLM 8). **E)** Correlations of regressors shown in Supplementary Figures 18b-c and 19b-c. Due to the high correlations between other and control uncertainties, we tested their time courses separately (ROI-GLMs 9 and 10) and together in one ROI-GLM 11. **F)** Correlations of regressors shown in Supplementary Figures 18d and 19d (ROI-GLM 12).

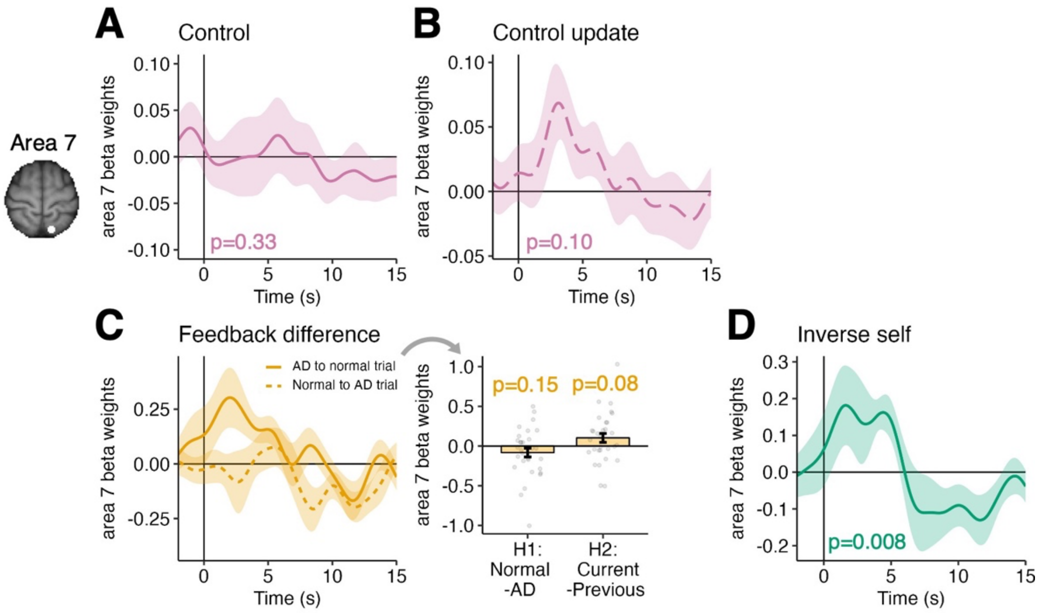

Supplementary Figure 14 (relating to Figure 6). Time courses of feedback difference, control and self in left area 7, time-locked to the outcome phase. A-B) We found no evidence that area 7 activity signals the inferred control or the control update at the time of outcome (control, t(30)=-0.98, p=0.33; control update, t(30)=1.69, p=0.10; ROI-GLM1). **C)** While the waveforms of feedback differences look more alike H2 than H1, we found no evidence in favour of either hypothesis in area 7 (H1, t(30)=-1.48, p=0.15; H2, t(30)=1.83, p=0.08; ROI-GLM2). This is unlike the SMG result that we found (Figure 6E). **D)** In the same GLM, we also tested for the inverse self. We indeed found that area 7 tracks the prior inverse self estimate at the time of outcome (inverse self, t(30)=2.82, p=0.008; ROI-GLM2). n=31 MRI, mean beta weights are plotted as lines, with s.e.m. as shaded intervals.

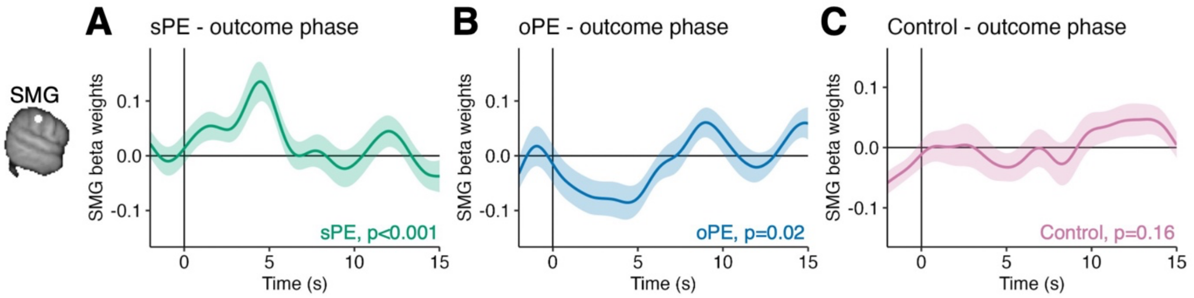

Supplementary Figure 15 (relating to Figure 7). Time course analyses of sPE, oPE and Control in the SMG. **A)** Due to the correlation of sPE and oPE, we also ran a separate time course analysis including both sPE and oPE in one ROI-GLM (ROI-GLM5). Here, we found a significant effect of sPE ($t(30)=3.83$, $p<0.001$). **B)** oPE, which was included in the same GLM, also still showed a significant effect ($t(30)=-2.41$, $p=0.02$). **C)** In ROI-GLM6, we tested for effects of tPE (plotted in main Figure 7C) and control in the outcome phase. We did not find that SMG represents the known control level ($t(30)=1.43$, $p=0.16$) in this phase of the task – the Self-Other phase – when participants had already been instructed about its level and did not need to infer it. n=31 MRI, mean beta weights are plotted as lines, with s.e.m. as shaded intervals.

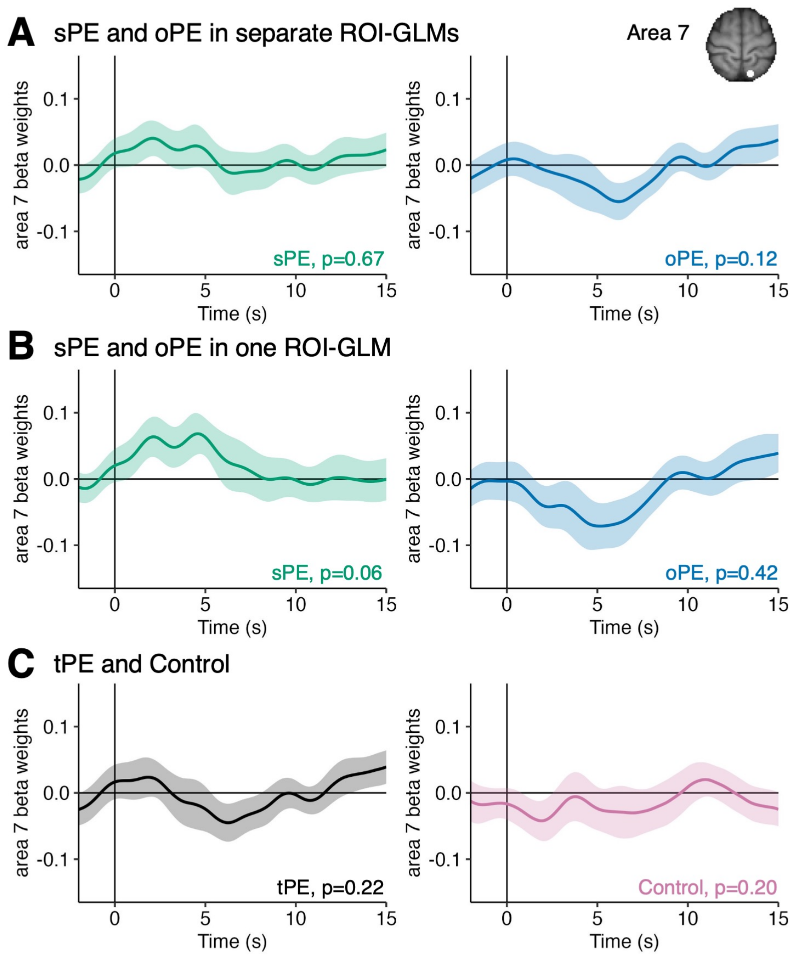

Supplementary Figure 16 (relating to Figure 7). Time courses of sPE, oPE, tPE and control in area 7 at the time of outcome, during the Self-Other phase. A) We did not find that area 7 BOLD activity is significantly modulated by sPE nor oPE when tested in separate GLMs (sPE, $t(30)=0.43$, $p=0.67$, ROI-GLM3; oPE, $t(30)=-1.58$, $p=0.12$, ROI-GLM4). **B)** Tested in one GLM, sPE and oPE remained not significant in area 7 (sPE, $t(30)=1.92$, $p=0.06$; oPE, $t(30)=-0.81$, $p=0.42$; ROI-GLM5). **C)** BOLD activity in area 7 was not significantly modulated by tPE nor the known control either (tPE, $t(30)=-1.26$, $p=0.22$; control, $t(30)=1.30$, $p=0.20$; ROI-GLM6). n=31 MRI, mean beta weights are plotted as lines, with s.e.m. as shaded intervals.

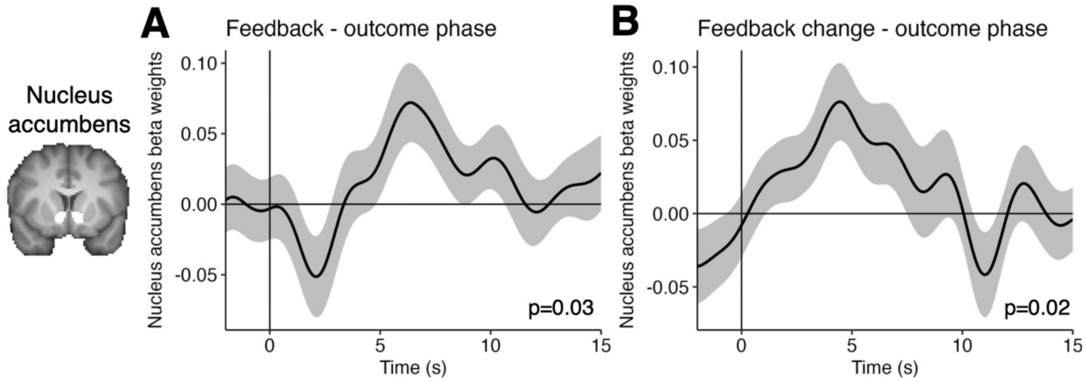

Supplementary Figure 17. Time courses of feedback and feedback change in the ventral striatum in the outcome phase. Previous studies have shown that the ventral striatum is sensitive to reward[1]. To test whether the ventral striatum also signals reward in our task, we ran a time course analysis on an anatomically defined bilateral mask of the nucleus accumbens (ROI-GLM7). **A)** We found that activity in the nucleus accumbens tracks the feedback that is being observed at the time of outcome (t(30)=2.23, p=0.03, ROI-GLM7). **B)** In the same GLM, we also found that the nucleus accumbens signals the change of the feedback (difference between current and last trial's feedback) at the time of outcome (t(30)=2.48, p=0.02, ROI-GLM7). Analyses were run on normal trials that followed normal trials, across Self-Other and Control-Other phase. The regressors show low correlations (r=0.23, see Supplementary Figure 13C). n=31 MRI, mean beta weights are plotted as lines, with s.e.m. as shaded intervals.

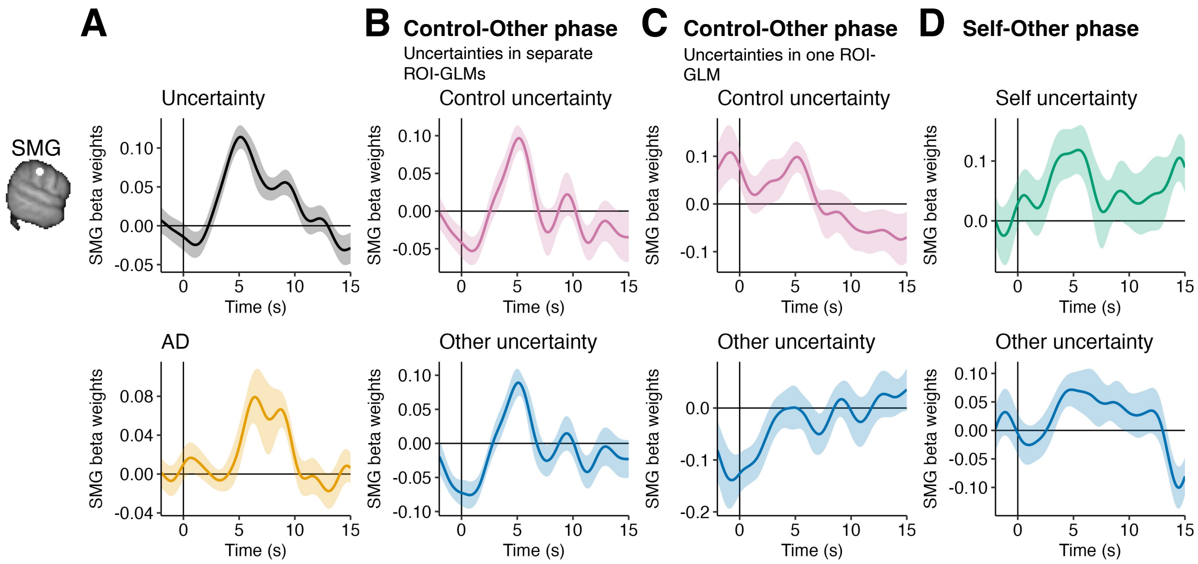

Supplementary Figure 18. SMG time courses of AD, (average) uncertainty and the uncertainties about self, other and control in the action phase. **A)** Time courses of uncertainty and AD across both Self-Other and Control-Other phases (ROI-GLM 8). Note that the SMG ROI was defined based on the overlapping clusters of the same regressors (uncertainty and AD) from the whole-brain analysis (shown in main Figure 5). Therefore, these time courses are purely for visualising this whole-brain effect in another way and no statistics have been performed on these regressors. **B-C)** Time courses of control and other uncertainties in the Control-Other phase. Since the regressors of control and other uncertainties showed high correlations (r=0.79), we visualised their time courses in separate ROI-GLMs first (panel B). When control and other uncertainties were tested in one ROI-GLM, due to their high correlations, their representations are weaker (panel C). **D)** Time courses of self and other uncertainties in the Self-Other phase. Regressor correlations are shown in Supplementary Figure 13D-F. n=31 MRI, mean beta weights are plotted as lines with s.e.m. as shaded intervals.

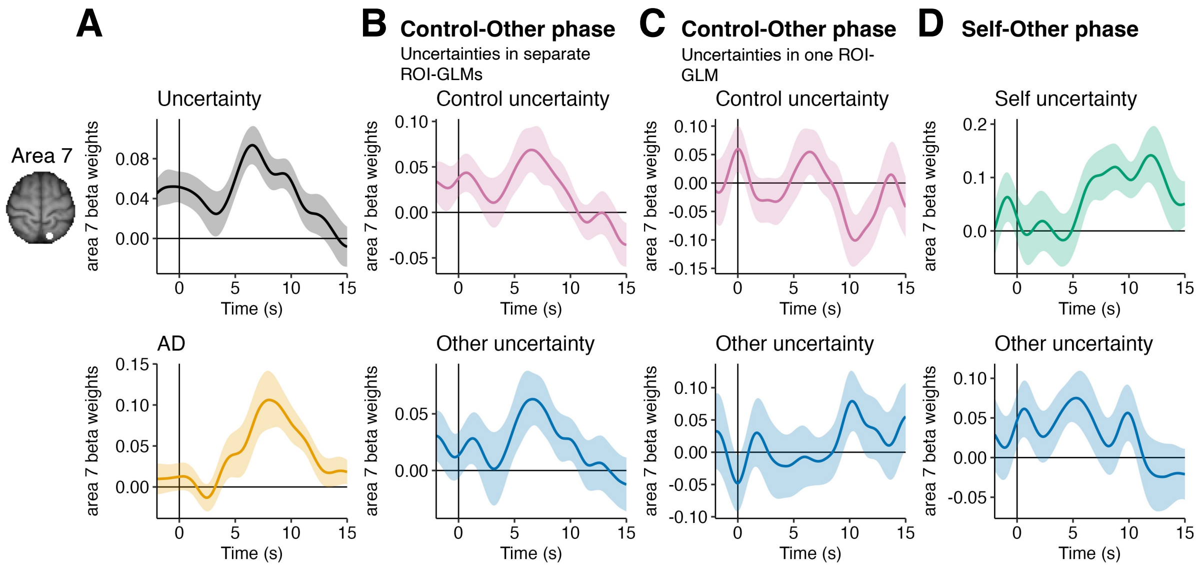

Supplementary Figure 19. Area 7 time courses of AD, (average) uncertainty and the uncertainties about self, other and control in the action phase. Since the area 7 ROI was defined based on whole-brain effects of uncertainty and AD, we did not perform statistics on these time courses to avoid circularity. This means these time courses are for illustration purposes only. **A)** Time courses of uncertainty and AD across Self-Other and Control-Other phases (ROI-GLM 8). **B-C)** Time courses of control and other uncertainties in the Control-Other phase, examined in separate and a joint ROI-GLM due to their high correlation. **D)** Time courses of self and other uncertainties in the Self-Other phase. Regressor correlations are displayed in Supplementary Figure 13D-F. n=31 MRI, mean beta weights are plotted as lines with s.e.m. as shaded intervals.

Supplementary tables

Supplementary Table 1 (relating to Figure 5). Peak coordinates of significant clusters in whole-brain fMRI analysis.

Contrast	Region	Peak coordinates x/y/z (in mm MNI space)	z value
Uncertainty	Lateral occipital cortex, superior division	-38, -80, 32	4.7
	Supramarginal gyrus (SMG)	-66, -24, 36	4.25
	Lateral occipital cortex, inferior division	-50, -66, 2	4.14
	Postcentral gyrus	-50, -38, 58	4.42
	Area 7	-12, -64, 66	3.94
	Superior parietal lobule	-38, -46, 64	3.78
	Intracalcarine Cortex	2, -84, 2	-4.77
	Superior temporal gyrus, posterior division	-56, -34, 4	-4.54
	Lateral orbitofrontal cortex (lOFC)	-50, 22, 0	-4.54
	Occipital pole	16, -90, 14	-4.17
	Dorsomedial prefrontal cortex (dmPFC)	-8, 52, 40	-3.92
	Temporal pole	-52, 12, -20	-3.9
Active disambiguation	Temporoparietal junction (TPJ)<br>- SMG subpeak (right):	52, -44, 34<br><br>SMG: 64, -34, 30	5.82<br><br>SMG: 4.70
	Dorsolateral prefrontal cortex (dlPFC)<br>- dmPFC subpeak:	Right: 28, 32, 34<br>Left: -44, 38, 28<br><br>dmPFC: 0, 40, 34	Right: 4.93<br>Left: 3.76<br><br>dmPFC: 4.50
	Precuneus	2, -50, 46	4.98
	SMG (left)	-64, -36, 32	5.04
	Lateral frontal pole	40, 52, 0	4.44
	Inferior frontal gyrus, pars opercularis, stretching into lOFC (left)	52, 18, -2	4.93
	Lateral occipital cortex, inferior division	Right: 40, -86, -8<br>Left: -48, -74, 4	Right: 4.08<br>Left: 3.98
	Lingual gyrus	-6, -78, -6	4.65
	Cerebellum, uvula	-26, -78, -24	4.80
	2 clusters in right lOFC (area 47o)	Cluster 1: 32, 22, -8<br>Cluster 2: 48, 24, -8	Cluster 1: 4.57<br>Cluster 2: 4.23
	Parahippocampal gyrus	32, -44, -6	4.82
	Cerebellum, tonsil	-38, -44, -44	3.82
	Middle temporal gyrus	-64, -46, -2	3.70
	Anterior cingulate cortex	10, 50, 12	4.13
	Cerebellum, anterior lobe	-32, -60, -32	4.11
	Area 4	Left: -42, -14, 50<br>Right: 44, -8, 58	Left: -4.61<br>Right: -4.52
	Supplementary motor area	0, -4, 60	-5.16

Family-wise error cluster corrected, $z > 3.1$, $p < 0.05$

Supplementary methods

Participants and recruitment

MRI sample. The analysed MRI data set originates from a larger sample with participants on a range of depression symptoms (findings to be reported elsewhere). To be included in the MRI study, participants were right-handed, aged 18-45 years, with normal or corrected to normal vision, and fluent in English. Participants were excluded if they had any contraindications for MRI scanning, current alcohol or drug dependency, current or past history of bipolar or psychosis disorder, or current use of psychoactive medication or recreational drugs. Interested individuals filled in a pre-screening online form hosted on Qualtrics. This online form included questions on demographics, MRI safety and a short depression questionnaire (Quick Inventory of Depressive Symptomatology, QIDS-SR)[2]. Participants were then invited to a pre-screening video call during which MRI safety was checked again, and during which part of the Structured Clinical Interview for DSM-5 Disorders was administered[3]. Participants were then invited to a single study visit. In this study, only participants with none or mild depressive symptoms (QIDS-SR total score < 11) were included.

Online sample. The study was advertised on the online platform Prolific (www.prolific.com). Participants were included into the study if they were aged 18-40 years old, had English as their first language, normal or corrected to normal vision, and at least 10 previous study submissions. Online participants could not move onto the main task if they failed the "system check" for the games. During this system check, participants practised the games and were instructed to do their best. For each of the games, participants had to produce five consecutive trials with sufficiently good game performance (defined as lower than 90% of largest absolute objective error from pilot data) to confirm that the games work correctly on their systems and that they paid attention. For each game, during task practice, participants had a maximum of 30 trials during which to produce those five good trials. Additionally, participants were excluded if after three attempts, they did not pass the multiple-choice test which checked their task understanding. After the first and second false attempt at this comprehension check, they were shown which questions they answered incorrectly and had to reread the instructions.

Experimental design

Post-task debrief questionnaires. After finishing the task, participants filled in a debrief questionnaire. One of the questions they answered was "In the study, how many times per game did you do badly on purpose?", which was answered on a Likert scale from "never", "less than once per game", 1, 2, …, 10, "More than 10 times per game". 5 acquired MRI participants (one excluded from analysis) did not fill in the debrief questionnaire because it was only added at a later stage. Online participants filled in a more extensive debrief questionnaire. One of the additional questions filled in only by the online sample was "If you did badly on purpose, how did it help you do each rating?", which for each rating (self, other, control) could be answered on a Likert scale from 1 – Not at all, 2, 3, 4, 5, 6 – Very much.

Mood ratings and psychiatric questionnaires. During the task, participants repeatedly rated their current mood. MRI participants also filled in a series of psychiatric questionnaires after the task and debrief questionnaire. Data from the mood ratings and psychiatric questionnaires were not analysed here and findings will be reported elsewhere.

Mapping of self performance. On every trial, participants' objective game performance was transformed into a self performance, according to the following logistic function:

$$\mathrm{Self(t)} = 100 * \left(1 - \left(1 + \mathrm{e}^{\mathrm{Slope(t)} * (\mathrm{Shift(t)} - \mathrm{Error(t)})}\right)^{-1}\right) \qquad \text{(S1)}$$

with

$$\mathrm{Slope(t)} = \frac{\ln\left((1-0.55)^{-1}-1\right)}{\mathrm{Error_{mean}} - \mathrm{Error_{min}}} \tag{S2}$$

and

$$\mathrm{Shift(t)} = \mathrm{Error_{mean}} + \ln\left(\left(1-\frac{\mathrm{Self_{level}}}{100}\right)^{-1} - 1\right) * \mathrm{Slope(t)}^{-1} \tag{S3}$$

where *Self(t)* is the transformed self performance (point score between 0 and 100) on a given trial *t*. *Error(t)* is the absolute objective performance error on trial *t*. In the rockslide game, for example, this was the absolute Euclidian distance (in pixels) between the target cross and the ball when participants pressed the button. If the participant pressed the button much too early or late, when the ball was not on the same slide as the target cross, their objective performance was always transformed to a *Self*=0 (active disambiguation trial, see Supplementary Figure 4). The parameter *Slope(t)* determines the steepness of the mapping curve and reflects how sensitive the self performance is to the objective errors. It is determined based on the mean ($Error_{mean}$) and smallest error ($Error_{min}$) from the previous five trials, excluding AD trials. On the first five trials of a game block, this was based on the pre-task training trials of the respective game. This staircasing procedure was introduced because some participants' errors drifted over time. Calibrating the slope to the participant's previous error range ensured that the mapped self performance has a constant variance across trials and is the same across participants. The *Shift(t)* parameter computes what error value is mapped onto the $Self_{level}$. $Self_{level}$ is the self performance level pre-determined by our schedules (see section of schedules), and is the mean self performance in the current task block. This is also the value that participants need to infer for their own self performance. The shift parameter depends on participants' mean errors across the previous five trials so that the $Self_{level}$ is given for participants' individual mean error. Calibrating the slope and shift parameters to the participants' recent error history ensured that the mapped self performance remained constant and centred around the $Self_{level}$. Supplementary Figure 2 illustrates the logistic function as well as the staircasing for an example participant.

Task instructions. Prior to the task, participants clicked through a series of task instruction screens including practice trials. This was then followed by a multiple choice quiz to check their understanding. In below screenshots, we obscured any face stimuli used with icons.

Our aim is to understand how people learn and how this relates to mood.
In this study you are a team captain and your task is to put a good team together. For this, you will play four games, together with other fictive players. For each game, you need to figure out how good you and the other players are to put the best team together.
You can earn extra money for doing this well.

In the Slingshot Game, the aim is to hit the target with the ball.
Next, we will check that this game works. Please do your best in this system check.

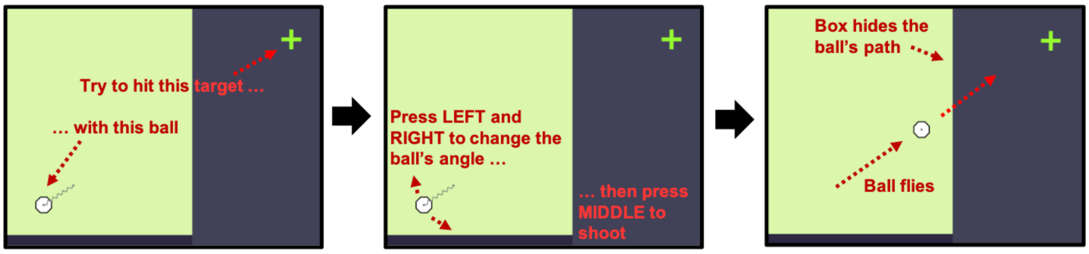

[practice trials for Slingshot game]

The next game is the Bouncing Ball Game:
Press LEFT to release the ball. As soon as you think it has passed the target flag, press LEFT again. Next, we will again check that this game works. Please do your best in this system check.

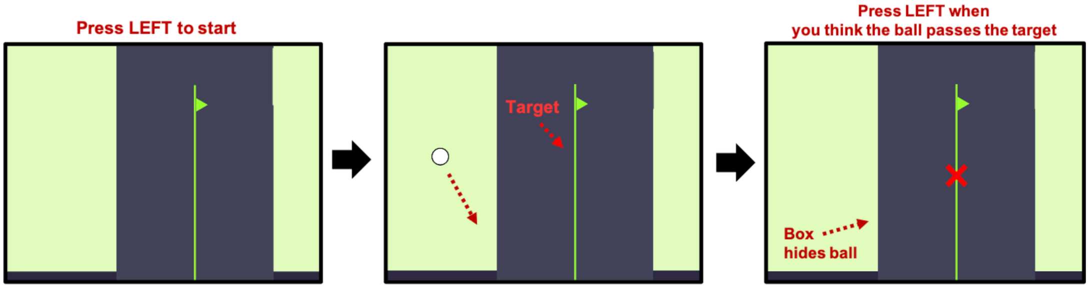

[practice trials for Bouncing Ball game]

The Rock Slide Game: Press LEFT to release the ball. Press LEFT when you think the ball hits the target. Please do your best in the next system check.

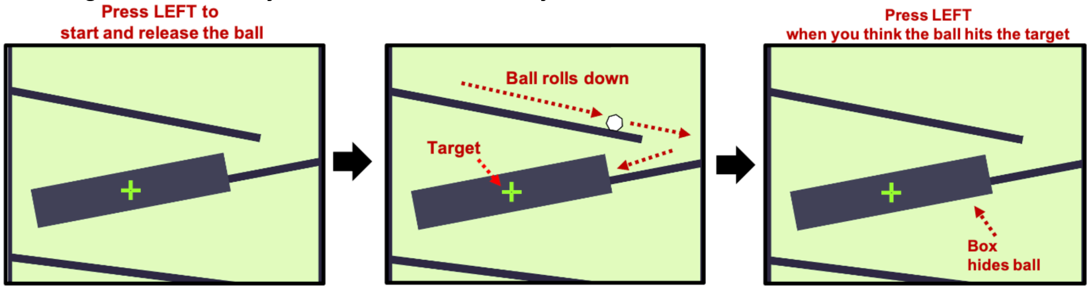

[practice trials for Rock Slide Game]

Finally, the Light Game: After you press LEFT, the circles light up one after another. Press LEFT when you think the target circle lights up. Please do your best in the following system check.

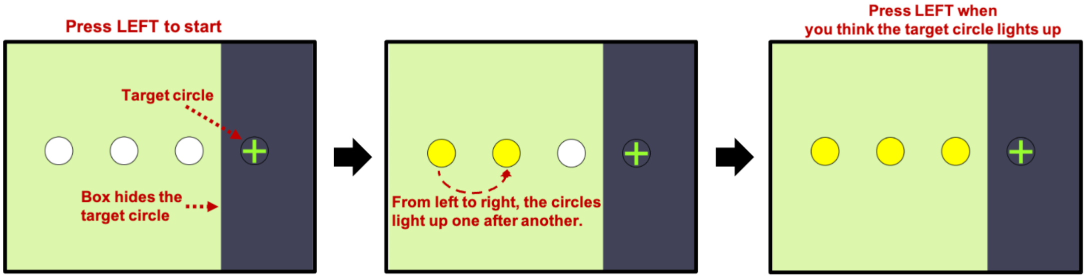

[practice trials for Light game]

You will play every game round together with another player
You will both play at the same time, but you will never see the other play.
Your aim as team captain is to pick good teammates for each game! Therefore, you will need to assess your own and the other player's performance in each game.
After each game round, a colored bar will show you how well you both did together.
> How well you do can change from game to game: In some, you will do better than in others.
> The players are computer-generated and based on real people. Therefore, they may get better or worse at the games over time. Also, some players will do better or worse than others.

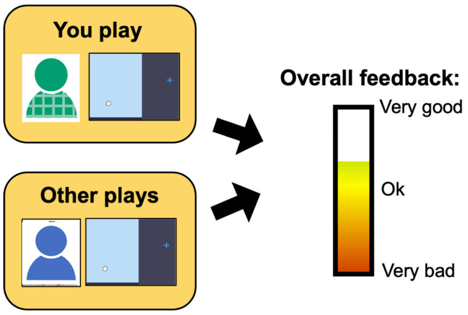

The feedback depends on how well you and the other do. Therefore, you influence only a part of the feedback.
> Sometimes, you and the other influence the feedback equally: How well you do matters as much as how well the other does.

> In other cases, you have more influence than the other.
> You can also have less influence than the other.
Example: A participant does well in the game and the other player does not. If the other has more influence, they will still get a low feedback together. (see below)
Another example: In football, you might do much worse than the rest of your team. Your team can still make many goals because you as a single player don't matter as much as the rest of the team.

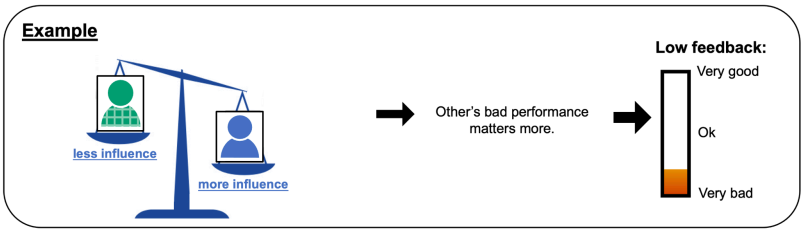

To put together your team, your task is to find out: (1) your influence over the feedback, (2) how well you do, and (3) how well the other player does.
You can do this by paying attention to the feedback after each game round. You can also try to change how you play the game to see how this affects the feedback.
Before each game round, you will make three ratings. Doing these ratings well is a crucial part of the study – you will get a bonus payment for doing them well and NOT for how well you play the games. The ratings will also help you to put a good team together after each game by selecting the best players and influence levels.
Press MIDDLE to practice one game round with ratings. Your ratings don't matter for now.

Press LEFT and RIGHT to rate. Press MIDDLE to submit

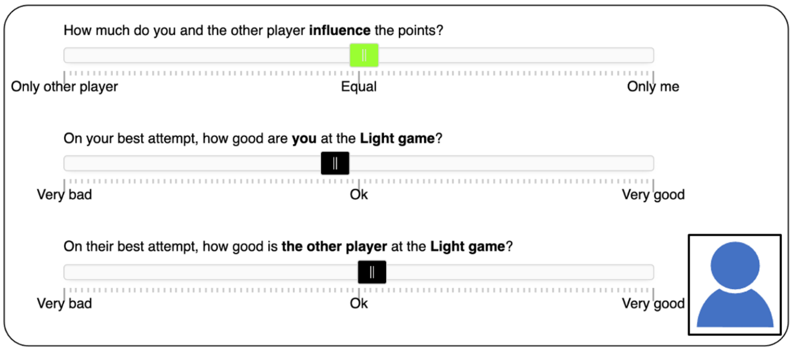

[practice trial]

At the end of each game, you will be asked to choose the team mates and distribute the influence within your team. The image below shows you what this will look like.

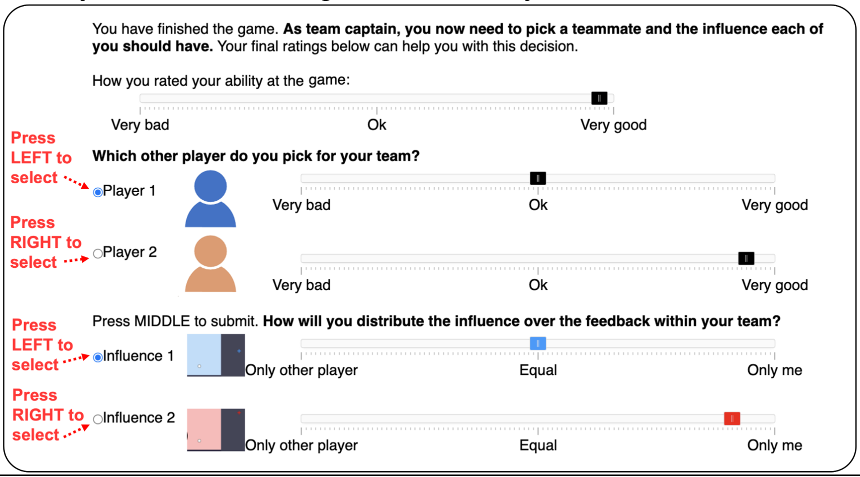

Sometimes, we will ask you about your mood. Then, please take a moment to think about how you feel. This is an important part of our study. Well done, that's it for the instructions.

Behavioural analyses

Accuracies of individual ratings and estimated feedback. If participants are presented with very good feedback, they should believe that either they themselves or the other player did very well (or even both). However, there is an infinite number of possible combinations of self, other and control that can all lead to the same feedback. For example, a low feedback could result from one's own performance being low and a high control for oneself, or from the other's performance being low and the other having a high level of control. This means that while participants' individual ratings (or beliefs) might not be accurate, their ratings in combination should closely match the ambiguous feedback.

Inferring self and other from active disambiguation. Just as control can be inferred in the Control-Other phase, self performance levels ("self") can be inferred from those trials during the Self-Other phase (see Supplementary 9A left). Here, self can be inferred from the difference between normal and AD trials, taking into account the known control level. We tested whether participants used this logic (equation S4) with a regression model (equation S5):

$$\text{Self} = \frac{\text{feedback difference}}{\text{Control}} \tag{S4}$$

$$\begin{gathered}\text{self}{\sim}\text{feedback difference} + \text{inverse control} + \\ \text{feedback difference} * \text{inverse control} + \text{previous self} + \\ (\text{feedback difference} + \text{inverse control} + \\ \text{feedback difference} * \text{inverse control} + \text{previous self}|\text{ID})\end{gathered} \tag{S5}$$

where *self* is the next trial's self rating. *Feedback difference* is the difference between normal and AD feedback, as observed on the current and previous trials. *Inverse control* is $\ln((\text{current trial's control level})^{-1})$, and *previous self* is the current trial's self rating. The hierarchical regression model was fit separately to participants' ratings, and to the respective control estimates of the active, ignorant and passive learning models. Similar to the equivalent control regressions, this regression was fit to switch trials only as this is when the feedback difference can be observed. We fit this regression to participants' self ratings as well as the self estimates of the active, passive and ignorant learning models. Results of this regression analysis are shown in Supplementary Figure 9B.

From the feedback equations it follows that participants could infer the other from the AD feedback accounting for the other's control. This is because on AD trials, the feedback shown is only based on the other's performance, weighted by the other's control. We tested whether participants used this logic (equation S6) using regressions fit to their other ratings (equation S7):

$$\text{Other} = \frac{\text{AD feedback}}{\text{Other control}} \tag{S6}$$

$$\begin{gathered}\text{other}{\sim}\text{AD feedback} + \text{inverse other control} + \\ \text{AD feedback} * \text{inverse other control} + \text{previous other} + \\ (\text{AD feedback} + \text{inverse other control} + \\ \text{AD feedback} * \text{inverse other control} + \text{previous other}|\text{ID})\end{gathered} \tag{S7}$$

where other is the next trial's other rating. *AD feedback* is the feedback observed on the current AD trial. *Inverse other control* is the inverse of the control that the other player exerts, i.e. $\ln((1\text{-Control})^{-1})$. This regression was fit only to AD trials as this is when the AD feedback can be observed, and fit separately to the Self-Other and the Control-Other phase. In the Self-Other phase, *inverse other control* is computed with the known control level, while it is computed based on the control ratings in the Control-Other phase. *Previous other* is the current trial's other rating, i.e. the rating just before observing the AD feedback. This regression was fit separately to participants'

other ratings, and the other estimates of the active, passive and ignorant learners. For the learning models, *inverse other control* was based on the control estimates from the respective model.

Effects driving AD trials and learning in the Control-Other phase. We examined the factors driving participants to perform AD and learn about their control rather than about the other in the Control-Other phase. We performed two analyses to address this (results are shown in Supplementary Figure 10). Firstly, we examined whether AD trials were driven more by the uncertainty about control than about the other player in the Control-Other phase. We ran the following hierarchical regression model across MRI and online samples to predict whether the current trial in the Control-Other phase is an AD trial or not:

$$\begin{aligned}\text{AD} \sim \text{control vs other uncertainty} + \text{trial number} + \\ (\text{control vs other uncertainty} + \text{trial number}|\text{ID})\end{aligned} \tag{S8}$$

with

$$\text{control vs other uncertainty} = 100 * \text{control uncertainty} - \text{other uncertainty} \tag{S9}$$

Where *control vs other uncertainty* is the difference between control and other uncertainty. Since control and other uncertainties are highly correlated (r=0.75), it is difficult to disentangle their effects when they are included as two separate regressors in one regression model. Therefore, we predicted the occurrence of AD trials by the difference between control and other uncertainty. This allowed us to quantify the relative contribution of control versus other uncertainty to AD trials. Intuitively, if the difference between control vs other uncertainty has a positive effect on AD, then AD trials were more driven by control than other uncertainty. If control vs other uncertainty has a negative effect, then AD trials were more driven by uncertainty about the other player. *Trial number* was included as a regressor of no interest. All trials in the Control-Other phase were included in this analysis and the data from online and MRI samples were combined.

Secondly, we investigated which factors contributed more to learning about control compared to the other player. We hypothesized that participants updated their beliefs about their control more than about the other player when control uncertainty was higher than other uncertainty, and following switch trials from AD to normal or vice-versa. Based on two of our previous findings, we also expected that AD trials contributed more to learning about control than the other player. These findings were that participants reported AD trials as more helpful to learn about control than other and that they did not learn about the other player from AD trials (Supplementary Figure 9E-F). We used the following regression analysis to test this:

$$\begin{aligned}\text{Control vs other updating} \sim \text{control vs other uncertainty} + \text{trial number} + \text{other changed} \\ * (\text{AD} + \text{switch trial}) + (\text{control vs other uncertainty} + \text{trial number} \\ + \text{other changed} * (\text{AD} + \text{switch trial})\ |\text{ID})\end{aligned} \tag{S10}$$

with

$$\text{control vs other updating} = 100 * |\text{control update}| - |\text{other update}| \tag{S11}$$

where *control vs other updating* is the difference between the absolute belief changes of control and the absolute belief changes of other. This measure allowed us to investigate whether participants updated their beliefs about the control more than the other player. We predicted this relative belief update by the difference between *control vs other uncertainty* (similar as in analysis above). In addition, we included whether the previous trial was an *AD trial*, or was a *switch trial* from AD to normal or vice-versa. The regressor *Other changed* denoted whether the current trial occurred when or after the other player changed performance unexpectedly. Furthermore, including interaction terms between other performance changes and AD and switch trials allowed us to investigate whether participants' relative learning about other and control changed before

and after the other player changed performance unexpectedly. Finally, we included *trial number* as a regressor of no interest. All trials except for the first phase trial of the Control-Other phase were included because on those trials, we could extract rating updates. Data from online and MRI sample were combined in this analysis.

Rating accuracies and AD over the course of the experiment. We examined whether people got better at the task over the course of the experiment. For this, firstly, we extracted their rating accuracies from the final trials of each task phase and task block (self from Self-Other phase only, control from Control-Other phase only, other averaged across both task phases). We then ran a set of ANOVAs predicting self, other and control accuracies by task block (1 to 4) as within and sample (online or MRI) as between-participant factors. Secondly, we computed the proportion of AD trials for each person across Self-Other and Control-Other phases in each task block. We then ran an ANOVA predicting the proportion of AD trials by task block (1 to 4) as within and sample (online or MRI) as between-participant factors. The results are shown in Supplementary Figure 11.

fMRI time course analysis on ROIs

Effects of AD and uncertainties at the time of action. We used time course analyses to follow-up on the whole-brain effects we found related to AD and uncertainty during the action phase (main Figure 5). Similar to the ROI-GLMs reported in the main text, we focussed our analyses on SMG and area 7 as ROIs. Firstly, we visualised the effects of AD and uncertainty across Self-Other and Control-Other phase as time courses. Importantly, note that this ROI-GLM uses the same regressors of interest and time-locking as the whole-brain analysis based on which SMG and area 7 were defined. Therefore, no statistical tests were performed on the resulting time courses and they are shown for visualisation purposes only. The results of the following ROI-GLMs are shown in Supplementary Figures 18b-d and 19b-d. We ran the following ROI-GLM 8 to the action phase across Self-Other and Control-Other phases:

$$\mathrm{BOLD} \sim \mathrm{AD} + \mathrm{uncertainty} + \mathrm{trial} + \mathrm{switch\ trial} + \mathrm{RT} + \mathrm{game\ type\ 1} + \mathrm{game\ type\ 2} + \mathrm{game\ type\ 3} \quad (\mathrm{S12})$$

where *AD* is a binary variable referring to whether the current trial is AD or not. *Uncertainty* is the averaged uncertainty across self, other and control. We included the same regressors of no interest as were included in the action phase of the whole brain analysis. *Trial* number was included to account for time in block. *Switch trial* was included to control for any neural effects related to switching from AD to normal or vice-versa. To control for reaction-time related effects, we included *RT* as a regressor of no interest. Finally, binary regressors for three of the four game types were included (*game type 1, game type 2 and game type 3*) to account for any differences between the games that participants played during the action phase. Note that here, we included three binary game type regressors so that the intercept of this ROI-GLM captured the fourth game type. The result of this ROI-GLM is shown in Supplementary Figures 18a and 19a. Regressor correlations are displayed in Supplementary Figure 13d.

Next, we examined whether SMG and area 7 represent the individual components of uncertainty. Specifically, we tested whether these ROIs tracked control and other uncertainties in the Control-Other phase, and self and other uncertainties in the Self-Other phase. Note that again, these regressors are not independent of the whole-brain effects based on which the ROIs were defined. Uncertainty was computed as the average across self and other uncertainties in the Self-Other phase, and control and other uncertainties in the Control-Other phase. While the regressors in the following ROI-GLMs are not identical to the whole-brain regressors, they correlate highly with each other (r>0.8, Supplementary Figure 13E-F). Therefore, we did not test the time courses for significance and the resulting time course plots are for illustration purposes only.

First, we investigated whether our ROIs represent control and other uncertainties at the time of action during the Control-Other phase. Since other and control uncertainties correlated highly with each other and with trial number (absolute r>0.6, Supplementary Figure 13e), we tested their time courses in separate regressions (ROI-GLM 9 and 10) and jointly in one regression (ROI-GLM 11).

$$\text{BOLD} \sim \text{control uncertainty} + \text{AD} + \text{switch trial} + \text{RT} + \text{game type 1} + \text{game type 2} + \text{game type 3} \quad \text{(S13)}$$

$$\text{BOLD} \sim \text{other uncertainty} + \text{AD} + \text{switch trial} + \text{RT} + \text{game type 1} + \text{game type 2} + \text{game type 3} \quad \text{(S14)}$$

$$\text{BOLD} \sim \text{control uncertainty} + \text{other uncertainty} + \text{AD} + \text{trial} + \text{switch trial} + \text{RT} + \text{game type 1} + \text{game type 2} + \text{game type 3} \quad \text{(S15)}$$

where *control uncertainty* and *other uncertainty* are the control and other uncertainty, extracted from the Active learner model. The other regressors included in these ROI-GLMs were parameters of no interest, similar to ROI-GLM 8.

Secondly, we investigated whether our ROIs represent the uncertainty about self and other at the time of action during the Self-Other phase. We tested their time courses in the following regression (ROI-GLM 12).

$$\text{BOLD} \sim \text{self uncertainty} + \text{other uncertainty} + \text{AD} + \text{trial} + \text{switch trial} + \text{RT} + \text{game type 1} + \text{game type 2} + \text{game type 3} \quad \text{(S16)}$$

where *self uncertainty* and *other uncertainty* are the uncertainties around self and other, extracted from the Active learner model. The other regressors were of no interest, similar to the ROI-GLMs above.

Supplementary notes

Control effect on self and other ratings. In the regression results shown in Figure 2E, we also find that overall, participants update their beliefs about the other (but not themselves) depending on the control level (main effect *1-Control* on other rating update: F(1,65)=16.02, p<0.001, η^2=0.20; *Control* on self-rating update: F(1,65)=0.88, p=0.35, η^2=0.01). The active learner showed a main effect of control on both self and other (*Control* on self, F(1,65)=69.38, p<0.001, η^2=0.51; *1-Control* on other, F(1,65)=68.33, p<0.001, η^2=0.51). Since we did not have hypotheses about this effect of control and the overall effect size is very small, we will not interpret it further.

Supplementary References

1. Wittmann, M.K., et al., *Predictive decision making driven by multiple time-linked reward representations in the anterior cingulate cortex.* Nature communications, 2016. **7**(1): p. 12327.
2. Rush, A.J., et al., *The 16-Item Quick Inventory of Depressive Symptomatology (QIDS), clinician rating (QIDS-C), and self-report (QIDS-SR): a psychometric evaluation in patients with chronic major depression.* Biological psychiatry, 2003. **54**(5): p. 573-583.
3. First, M.B., et al., *SCID-5-CV: Structured clinical interview for DSM-5 disorders: Clinician version.* (No Title), 2016.
