# Supplementary material for: Active disambiguation guides inferring controllability and cause in social interactions
Source: Nat Commun. 2025 Dec 22;16:11568. doi: 10.1038/s41467-025-67853-8 (PMC12748874; doi:10.1038/s41467-025-67853-8)
Supplement: Supplementary file 2 — Reporting Summary [file 41467_2025_67853_MOESM2_ESM.pdf]

nature portfolio

Corresponding author(s): Lisa Spiering

Last updated by author(s): Nov 11, 2025

Reporting Summary

Nature Portfolio wishes to improve the reproducibility of the work that we publish. This form provides structure for consistency and transparency in reporting. For further information on Nature Portfolio policies, see our Editorial Policies and the Editorial Policy Checklist.

Statistics

For all statistical analyses, confirm that the following items are present in the figure legend, table legend, main text, or Methods section.

n/a	Confirmed	
☐	☒	The exact sample size (n) for each experimental group/condition, given as a discrete number and unit of measurement
☐	☒	A statement on whether measurements were taken from distinct samples or whether the same sample was measured repeatedly
☐	☒	The statistical test(s) used AND whether they are one- or two-sided<br>*Only common tests should be described solely by name; describe more complex techniques in the Methods section.*
☐	☒	A description of all covariates tested
☐	☒	A description of any assumptions or corrections, such as tests of normality and adjustment for multiple comparisons
☐	☒	A full description of the statistical parameters including central tendency (e.g. means) or other basic estimates (e.g. regression coefficient) AND variation (e.g. standard deviation) or associated estimates of uncertainty (e.g. confidence intervals)
☐	☒	For null hypothesis testing, the test statistic (e.g. F, t, r) with confidence intervals, effect sizes, degrees of freedom and P value noted<br>*Give P values as exact values whenever suitable.*
☐	☒	For Bayesian analysis, information on the choice of priors and Markov chain Monte Carlo settings
☐	☒	For hierarchical and complex designs, identification of the appropriate level for tests and full reporting of outcomes
☐	☒	Estimates of effect sizes (e.g. Cohen's d, Pearson's r), indicating how they were calculated

Our web collection on statistics for biologists contains articles on many of the points above.

Software and code

Policy information about availability of computer code

Data collection	Behavioural data was collected using java script and JATOS (Lange et al., 2015). Functional magnetic resonance imaging (fMRI) data were acquired using a Siemens Prisma 3T MRI scanner.
Data analysis	We analysed data using R (4.2.1), FSL (6.00) and Matlab (2021a). The computational models were fit using cmdstanr (0.5.3), and the Bayesian regression analyses were performed using brms (2.17.0) built on Stan. The code can be found at https://github.com/lisaspiering/cause-controllability

For manuscripts utilizing custom algorithms or software that are central to the research but not yet described in published literature, software must be made available to editors and reviewers. We strongly encourage code deposition in a community repository (e.g. GitHub). See the Nature Portfolio guidelines for submitting code & software for further information.

Data

Policy information about availability of data

All manuscripts must include a data availability statement. This statement should provide the following information, where applicable:

- Accession codes, unique identifiers, or web links for publicly available datasets
- A description of any restrictions on data availability
- For clinical datasets or third party data, please ensure that the statement adheres to our policy

The data used in this study are available at https://github.com/lisaspiering/cause-controllability (for behavioural and MRI data), and the MRI data can also be found on neurovault (accession code: https://identifiers.org/neurovault.collection:21965).

Research involving human participants, their data, or biological material

Policy information about studies with human participants or human data. See also policy information about sex, gender (identity/presentation), and sexual orientation and race, ethnicity and racism.

Reporting on sex and gender	We asked for participants' self-reported gender at the time of data collection. Out of a total of 31 MRI participants, 22 self-reported as female. Out of a total of 36 online participants, 21 self-reported as female and 1 as diverse. We do not include further analysis of gender because it was not applicable to our research questions.
Reporting on race, ethnicity, or other socially relevant groupings	We did not collect data on race or ethnicity because it was not applicable to our research questions.
Population characteristics	The final samples included 31 MRI participants (22 female, aged 18–33 years), and 36 online participants (21 female, 1 diverse, aged 18–40 years).
Recruitment	MRI participants were recruited via email circulation on Oxford University mailing lists, study flyers, social media platforms and the departmental database. The online study was advertised on the online platform Prolific (www.prolific.com).
Ethics oversight	The study was approved by the Medical Sciences Inter-Divisional Research Ethics Committee (MSD-IDREC, R40628/RE005 for MRI, R54722/RE008 for online) at the University of Oxford.

Note that full information on the approval of the study protocol must also be provided in the manuscript.

Field-specific reporting

Please select the one below that is the best fit for your research. If you are not sure, read the appropriate sections before making your selection.

☐ Life sciences ☒ Behavioural & social sciences ☐ Ecological, evolutionary & environmental sciences

For a reference copy of the document with all sections, see nature.com/documents/nr-reporting-summary-flat.pdf

Behavioural & social sciences study design

All studies must disclose on these points even when the disclosure is negative.

Study description	Data are quantitative experimental data. The MRI study involves imaging data from a 50min fMRI scan, behavioural data during the scan, and post-task debrief questionnaire data. The online study includes behavioural and post-task debrief questionnaire data.
Research sample	The MRI sample included 31 students, staff and public members in and around Oxford (22 female, aged 18–33 years).<br>The online sample included 36 participants (21 female, 1 diverse, aged 18–40 years).
Sampling strategy	The MRI data set originates from a larger sample with participants on a range of depression symptoms (findings to be reported elsewhere). In this study, only participants with no or mild depressive symptoms were included (assessed with the QIDS-SR questionnaire, Rush et al., 2003). No statistical methods were used to pre-determine sample sizes. Our sample size was chosen as larger to those reported in previous publications (e.g., Trudel et al., Nature Human Behaviour, 2021; Holton et al., Nature Human Behaviour, 2024). The sample was screened (e.g. for MRI safety, see other in-/exclusion criteria in methods) and thus was not representative of the general population, but suitable for investigating our research questions. The in-/exclusion criteria reduce heterogeneity but may bias results toward cognitively high-functioning, highly compliant individuals who are comfortable with computer-based tasks and performing them inside an MRI scanner.
Data collection	After providing written informed consent, MRI participants participated in a practice session outside the MRI scanner with the researcher in the room. MRI participants then performed the task inside the MRI scanner, in a separate room to the researcher and radiographers. All data was recorded with a computer using javascript and JATOS, and the MRI scanner. Since we used a within-participant study design, participants were not allocated to different experimental conditions and so the researcher was not blinded to them or the study hypotheses. Online participants were recruited via Prolific and tested remotely.

Timing	MRI participants were collected from October 2021 to May 2023 (with data collection limited by shortages in scanner availability due to the pandemic). Online participants were collected in two batches in August 2021 and July 2022.
Data exclusions	36 healthy participants took part in the fMRI experiment and 69 participants took part in the online experiment. 2 MRI participants were excluded because they misunderstood how they should rate their own performance in the games. 3 additional MRI participants were excluded because they performed fewer than 3 active disambiguation trials in the Control-Other phase. For the online sample, 3 participants were excluded because they took breaks longer than 5 min during the task blocks, and 9 participants were excluded because they misunderstood how to rate their own performance. In addition and for almost all of our analyses (except the correlation analysis shown in Figure 3d), we excluded 30 online participants who did not perform AD in the task or reported never having done AD in the post-task debrief questionnaire. The final samples included 31 MRI participants and 36 online participants .
Non-participation	See "data exclusions"
Randomization	Participants were not allocated into experimental groups.

Reporting for specific materials, systems and methods

We require information from authors about some types of materials, experimental systems and methods used in many studies. Here, indicate whether each material, system or method listed is relevant to your study. If you are not sure if a list item applies to your research, read the appropriate section before selecting a response.

Materials & experimental systems

n/a	Involved in the study	
☒	☐	Antibodies
☒	☐	Eukaryotic cell lines
☒	☐	Palaeontology and archaeology
☒	☐	Animals and other organisms
☒	☐	Clinical data
☒	☐	Dual use research of concern
☒	☐	Plants

Methods

n/a	Involved in the study	
☒	☐	ChIP-seq
☒	☐	Flow cytometry
☐	☒	MRI-based neuroimaging

Plants

Seed stocks	n/a
Novel plant genotypes	n/a
Authentication	n/a

Magnetic resonance imaging

Experimental design

Design type	Event-related design
Design specifications	Participants completed one study session with the task comprising 144 trials, divided into four blocks. Trials were reaction time (RT)-paced. Between trials, a short interval was included (inter-trial interval, ITI), during which a fixation cross was shown. For the MRI sample, the duration of the ITI was drawn from a Poisson distribution with a range of 2 to 7s, in order to decorrelate variables of interest between trial phases. For the online sample, the ITI had a fixed duration of 500ms.
Behavioral performance measures	We recorded participants' button presses and response times. Performance was quantified as how accurately participants did the ratings and they received a bonus payment for this.

Acquisition

Imaging type(s): Functional and structural

Field strength: 3T

Sequence & imaging parameters: Imaging data were acquired with a Siemens Prisma 3T MRI using a 32-channel head-coil. T1 weighted structural scans were obtained with repetition time (TR) = 1,900ms, echo time (TE) = 3.96ms and 1×1×1mm voxel size. Functional images were acquired using a multiband T2*-weighted echo planar imaging sequence with acceleration factor of two, and with an oblique angle of 30° to the posterior commissure (PC)– anterior commissure (AC) line to reduce signal dropout in orbitofrontal regions (Deichmann et al., 2003). Other acquisition parameters included 2.4×2.4×2.4 mm voxel size, TE = 30ms, TR = 1,360ms, 60° flip angle, a 240mm field of view and 66 slices per volume. Bias correction was applied directly to the scan.

Area of acquisition: Whole brain

Diffusion MRI: ☐ Used ☒ Not used

Preprocessing

Preprocessing software: Imaging data was analysed using FMRIB's Software Library (FSL, 6.00). Preprocessing stages included motion correction, correction for spatial distortion by applying the fieldmap, brain extraction, spatial smoothing (using a full-width at half-maximum of 3 mm), and high-pass temporal filtering (Gaussian-weighted least-squares straight line fitting, with sigma=22.5s).

Normalization: Functional images were first co-registered to an individuals' T1 structural image and then nonlinearly registered to the Montreal Neurological Institute (MNI) template using 12 degrees of freedom.

Normalization template: The MNI152 template was used, as standard in FSL.

Noise and artifact removal: Preprocessing stages included motion correction, correction for spatial distortion by applying the fieldmap, brain extraction, spatial smoothing (using a full-width at half-maximum of 3 mm), and high-pass temporal filtering. Cardiac and respiratory data were processed using FSL's Physiological Noise Modelling (PNM) tool to model the effects of physiological noise in the MRI data (Brooks et al. 2008).

Volume censoring: No volume censoring was used.

Statistical modeling & inference

Model type and settings: We used a univariate model. First, we estimated contrasts for each participant with a first-level analysis using FSL FEAT. Temporal derivatives, standard motion correction parameters and physiological noise regressors were included in the model. We used a second-level analysis (FSL FLAME1) to combine data across subjects, with cluster-correction threshold of $z > 3.1$ and $p < 0.05$.

Effect(s) tested: We used a single GLM for whole brain analysis. We tested for effects of uncertainty and active disambiguation during the action phase (Fig. 5). The full model is described in the methods including the regressors entered in the analysis (see "fMRI whole brain analysis").

Specify type of analysis: ☐ Whole brain ☐ ROI-based ☒ Both

Anatomical location(s): We selected ROIs in the left SMG and area 7 on the basis of activity peaks from independent regressors (uncertainty and AD at the time of action) identified from the whole-brain analysis. We then tested whether these regions are involved in feedback processing at the time of outcome. In the supplementary material, we used an anatomically defined bilateral mask of the nucleus accumbens (Khalighinejad et al., Nature Communications, 2021).

Statistic type for inference (See Eklund et al. 2016): Whole-brain clusters were corrected with family family-wise error cluster correction of $z>2.3$ and $p<0.05$.

Correction: Family-wise error cluster correction ($z > 3.1$ and $p < 0.05$).

Models & analysis

n/a	Involved in the study
☒	☐ Functional and/or effective connectivity
☒	☐ Graph analysis
☒	☐ Multivariate modeling or predictive analysis
